# Conformity to popular, not average, opinions: Models, data, and evolution

**DOI:** 10.1073/pnas.2530712123

**Published:** 2026-06-18

**Authors:** Kaleda K. Denton, Marcus W. Feldman, Jonathan F. Johannemann

**Affiliations:** ^a^ Santa Fe Institute, Santa Fe, NM 87501; ^b^ Department of Biology, Stanford University, Stanford, CA 94305; ^c^Independent researcher, San Francisco, CA 94158

**Keywords:** conformity, French-DeGroot model, opinion dynamics, networks, wisdom of the crowd

## Abstract

In assessing how individuals in a network estimate a quantity, it is often assumed that they take an average or weighted average of the estimates of others they observe, as in the French–DeGroot learning model. However, individuals may actually copy clusters of similar opinions among their peers, a process that we call conformity. For example, if ten individuals estimate a quantity to be 0 and ten individuals guess 100, then although the average estimate is 50, one may conform by choosing either 0 or 100. Here, we a) extend a recent model of conformity to include both personal beliefs and social information, b) evaluate this model and two models of French–DeGroot averaging on human decision-making data, and c) simulate evolutionary dynamics of each model under different kinds of population structure, including static and adaptive networks. Under many of the conditions we analyze, the conformity model provides a significantly better fit to the data than either of the French–DeGroot models. In addition, evolutionary simulations reveal considerable differences among the models; for example, compared to the French–DeGroot models, the conformity model can produce faster shifts in populations toward “extreme” opinions—where individuals’ estimates of a quantity are either very large or very small—and can reduce individuals’ average estimation accuracy. These findings have implications for research on consensus formation, polarization, and wisdom of crowds.

## Significance

A continuous trait contains infinitely many variants on a spectrum, such as a spectrum of behaviors or ideologies. “Conformity” to such traits has been defined as the preference for the mean variant, even if this mean is not close to any individual variant (e.g., if half of the population falls on the far right and far left of a spectrum, respectively, the mean is in the center). Here, we define conformity as the preference for clusters of common variants, not average variants. Compared to trait-averaging models, this conformity model provides a better fit to empirical data on human decision-making under many conditions, and in simulations, it often produces different population-level outcomes such as faster shifts toward poles of a spectrum.

How individuals form opinions, and how the opinions of many individuals aggregate to produce emergent group-level properties, are questions explored in fields such as cultural evolution, animal behavior, statistical physics, and artificial intelligence. Conceptions of “individuals,” “opinions,” and “emergent properties” differ depending on the field, but the underlying mathematical models can be similar. For example, in cultural evolution (1), an individual may be a person, an opinion may be a political view, and emergent group-level properties might include norms, fads, or polarization. In statistical physics, an individual may be an electron, its state (analogous to “opinion”) may be its spin, and emergent properties may include phase transitions in magnetizable materials (2).

In both physical and social systems, two commonly studied mechanisms for acquiring opinions are conformity and anticonformity. In broad terms, conformity refers to the tendency to adopt popular opinions and anticonformity refers to the opposite tendency, although specific definitions differ by field and by the system studied. Empirical researchers have documented these biases in humans (3–5) and nonhuman animals such as nine-spined sticklebacks (6), great tits (7), and fruit flies (8), among possibly many more (see table 1 in ref. 9). Conformity has also been shown in large language models when questions across a wide variety of knowledge domains are answered (10, 11). In statistical physics, conformity is analogous to ferromagnetic interactions and anticonformity is analogous to antiferromagnetic interactions (2, 12).

Theoretical studies of conformity and anticonformity have largely focused on binary choices, i.e., “adopt A” vs. “adopt B” (2, 12–26). These binary choices may be, for example, a pro vs. con view, an adaptive vs. maladaptive behavior, or an up vs. down spin of an electron. Other models of conformity and anticonformity have included more than two discrete traits (2, 12, 27–33). However, in comparison with the rich literature on discrete traits, fewer models of conformity and anticonformity have focused on continuously varying traits with infinitely many variants. Examples of such traits include the level of behavior on a spectrum, the proportion of time spent on a task, and the estimation of a numerical quantity such as a fraction or correlation.

Among the studies that have modeled conformity to continuous traits, some have defined conformity as a preference for the average trait value among observed individuals (34, 35). Similarly, in other studies that do not use the term “conformity,” individuals are assumed to adopt the average of others’ traits (36), or a weighted average of their own and others’ traits (37). Commonly used models that incorporate trait averaging include the French–DeGroot model (38, 39) (also known as the “DeGroot model,” defined in Section 1.1) and the Hegselmann–Krause model (40) (see also ref. 41).

In particular, the French–DeGroot model has been considered “the foundational opinion diffusion model and most influential non-Bayesian model” (42), and it is widely used due to its analytical tractability (43). Risco et al. (44) state that “empirical evidence heavily supports DeGroot updating. Various papers confront it against Bayesian learning in an experimental setting, concluding that it approximates better people’s information aggregation rules.” The French–DeGroot model has been used and extended by theorists to investigate a diverse array of phenomena such as consensus formation (45, 46), polarization (47), information diffusion (48), emergency decision-making (49), and the wisdom of crowds (37).

Recently, Heinrich Mora et al. (50) introduced a model of conformity to continuous traits that did not rely on averaging the observed traits. They argued that the mean of many opinions may not be close to the most “popular” opinion and therefore may not be preferred by conformists, “especially if the mean is affected by outliers or if the population is polarized” (50, p. 8]. For example, suppose ten individuals independently estimate a numerical quantity that ranges between 0 and 100. There may be an outlier; e.g., one individual estimates 0 and nine individuals estimate 100, producing an average of 90. A conformist observing these responses may prefer to discard the outlier and choose the most popular answer, 100, as opposed to the average. Or, there may be polarization; e.g., five individuals estimate 100 and five estimate 0, producing an average of 50. In this case, a conformist may prefer to choose either 100 or 0, similar to the answers of others, rather than 50. Thus, instead of defining the most popular opinion as the mean, Heinrich Mora et al. (50) defined the most popular opinion as that which is most similar to the opinions of others (Section 1.2). Hereafter, we will refer to their model as “conformity” and other models that rely on opinion averaging as averaging models.

In addition to the French–DeGroot and conformity models, there is a vast opinion dynamics literature with many other kinds of opinion updating rules. For example, several models besides the conformity model overcome one of the main limitations of French–DeGroot averaging; namely that if individuals’ opinions are highly polarized, then the average is not reflective of the most popular opinion. Some models have introduced a “threshold” or “confidence level” ε such that individuals only adopt opinions that differ from their own by no more than ε (40, 51). However, these models do not capture the phenomenon, shown extensively in empirical studies, of individuals abandoning their personal opinions when others’ opinions differ significantly from theirs (52–54). For example, if your personal opinion were that 0% of jelly beans in a jar were red (and 100% were orange) but five others said that 100% of the jelly beans in the jar were red, then you might switch your estimate to 100% despite the five others’ guesses being very different from your own (i.e., not within a small threshold ε of your initial guess). In this study, we focus on the French–DeGroot model and the conformity model of ref. 50 rather than threshold models.

In ref. 50, an individual’s choice of cultural variant on a spectrum was based solely on the choices of others that were observed (social information), without personal information that could affect the outcome. However, in many real-world settings, individuals base their opinions on both the opinions of others and their own beliefs. Therefore, in Section 1.3, we generalize the model of ref. 50 to allow both social and personal information to affect individuals’ decisions.

We evaluated the performance of this conformity model, as well as two commonly used versions of the French–DeGroot model, on human decision-making data from Almaatouq et al. (37). These researchers conducted an experiment in which participants 1) estimated the correlation in a scatter plot, which could range between 0 and 1; 2) viewed the answers of up to three other participants; and 3) had the option to revise their estimates based on this social information or stick with their original estimates. Almaatouq et al. also included a simulation in which agents chose a number between 0 and 1 (their “estimate”), then updated this number according to a weighted average of others’ estimates and their own. Specifically, the agents were placed in a network and the authors implemented a two-stage French–DeGroot model, where both the opinions of agents’ immediate neighbors and the opinions of these neighbors’ neighbors could influence their decisions. A one-stage French–DeGroot model, on the other hand, only takes into account an agent’s immediate neighbors.

Almaatouq et al. (37) did not apply their mathematical model to experimental data, but used it to assess the generalizability of their conclusions. However, it is also interesting to determine the extent to which the two-stage French–DeGroot model, as well as the commonly used one-stage French–DeGroot model and the conformity model, fit the data from this behavioral experiment. Here, under many of the conditions that we analyzed, the conformity model was found to provide a better fit to the data of ref. 37 than either of the French–DeGroot models.

Establishing which of these three models provides the best fit to experimental data is especially important if the models produce different theoretical predictions. Thus, we explored the effects of the one-stage French–DeGroot, two-stage French–DeGroot, and conformity models on population dynamics in different kinds of networks. We found that the conformity model often produced different dynamics from the two French–DeGroot models. Therefore, reevaluating theoretical research on opinion dynamics in the context of conformity rather than opinion averaging may produce interesting new insights.

## 1. Models

### 1.1. Overview of the French–DeGroot Model.

In the French–DeGroot model (38, 39), the weight that individual i places on individual j’s opinion is denoted by $T_{i,j}$, an entry in the row-stochastic “trust matrix” T. For example, suppose individual i=1 samples the opinions of n other individuals, j=2⋯n+1, and places equal weight on each of these peers’ opinions. As the focal individual does not know the opinions of other individuals (j>n+1), the weight placed on their opinions is zero. In addition, let the focal individual (i=1) have α times as much trust in its own, personal judgment relative to 1 for the opinion of any other individual in its sample; in other words, for any j in 2,⋯,n+1, $T_{1,1}=\alpha T_{1,j}$. Then, if the population size is N, the first row in T is

$$\begin{array}{c} \begin{array}{cc} & \left[\begin{array}{ccccccc} T_{1,1} & T_{1,2} & \cdots & T_{1,n+1} & T_{1,n+2} & \cdots & T_{1,N} \end{array}\right]\\ & =\left[\begin{array}{ccccccc} \frac{\alpha }{\alpha +n} & \frac{1}{\alpha +n} & \cdots & \frac{1}{\alpha +n} & 0 & \cdots & 0 \end{array}\right]. \end{array} \end{array}$$

If α=∞, individuals place 100% of the weight on their own, personal opinions; the opinions of others do not affect them.

At time t, let the vector of personal opinions of each of the N individuals in the population be denoted by $u^{\left(t\right)}$. Then, given a trust matrix $T^{\left(t\right)}$, the opinions in the population are updated according to

[1] $$\begin{array}{cc} u^{\left(t+1\right)} & =T^{\left(t\right)}u^{\left(t\right)}. \end{array}$$

Alternatively, in a two-stage French–DeGroot process, the trust matrix is applied twice so that the connections of one’s connections also influence one’s opinion:

[2] $$\begin{array}{cc} u^{\left(t+1\right)} & ={\left(T^{\left(t\right)}\right)}^{2}u^{\left(t\right)}. \end{array}$$

Almaatouq et al. (37) used the two-stage French–DeGroot process to model social interaction (see their *SI Appendix*, Eq. **S1**).

### 1.2. Overview of the Conformity Model.

A full evolutionary model is given in ref. 50. Here, we concentrate on one part of that model: For a given focal individual that has sampled n other individuals’ opinions, how does the focal individual form an opinion? For the moment, assume that the focal individual’s opinion depends only on the opinions of the n others, and not on its personal belief.

Assume that the focal individual has sampled n≥3 individuals (not including itself), as in refs. 13, 20, and 50. Three individuals is the smallest number required to produce a majority and minority opinion and therefore to allow conformity. The case of n<3 will be discussed later.

Without loss of generality, assume that an individual’s opinion can take values between 0 and 1, as in refs. 37 and 50. Denote the n observed individuals’ opinions by $x=(x_{1},x_{2},\cdots ,x_{n})$. For example, n=4 observed individuals’ estimates of a scatterplot correlation may be x=(0.5,0.75,0.71,0.9). Then, the model proceeds in three steps.

**Step 1:** for each opinion $x_{i}$ in x, a measure of closeness to the other opinions is calculated. Specifically, this measure, called k-dispersal, is defined as the sum of the k shortest absolute distances between $x_{i}$ and the other $x_{j}$ in x, where j≠i and k<n (see definition 2 in ref. 50).

For example, let k=2. If x=(0.5,0.75,0.71,0.9), then the k-dispersal of 0.5 is |0.5−0.71|+|0.5−0.75|=0.46. The k-dispersal of 0.71 is |0.71−0.75|+|0.71−0.9|=0.23. This tells us that compared to the number 0.5, the number 0.71 has a lower dispersal from, or is closer to, the other numbers in the sample.

**Step 2:** the focal individual assigns a probability to adopting each of the n opinions $x_{1},\cdots ,x_{n}$ based on their k-dispersals. If the focal individual is a conformist, then more densely clustered opinions (i.e., those with lower k-dispersals) have higher probabilities of adoption than less densely clustered opinions (i.e., those with higher k-dispersals). Therefore, in the above example, a conformist would have a higher probability of choosing 0.71 than 0.5. An anticonformist, on the other hand, prefers less densely clustered opinions over more densely clustered opinions and would have a higher probability of choosing 0.5 than 0.71.

The formula specifying the probabilities that an observer adopts any opinion $x_{i}$ in $x=(x_{1},x_{2},\cdots ,x_{n})$, for a given dispersal parameter k, is

[3] $$\begin{array}{cc} P_{k}\left(x_{i}|x\right) & =\frac{1}{n}+\frac{g_{i,k}\left(x\right)d_{k}\left(x\right)}{n}, \end{array}$$

as in Eq. **1** of ref. 50. The function $g_{i,k}\left(x\right)$, given in Eq. **4** below, takes a positive value if variant $x_{i}$ is closer to the other variants in the sample, i.e., has a lower k-dispersal than the average k-dispersal of the variants in the sample. Conversely, $g_{i,k}\left(x\right)$ is negative if variant $x_{i}$ is farther from the other variants in the sample, i.e., has a higher k-dispersal than the average. The coefficient $d_{k}\left(x\right)$ determines the level of (anti)conformity. If $d_{k}\left(x\right)$ is positive, then individuals exhibit conformity: More densely clustered variants, i.e., those with positive $g_{i,k}\left(x\right)$, are adopted with probability greater than $\frac{1}{n}$ by Eq. **3**, whereas less densely clustered variants with negative $g_{i,k}\left(x\right)$ are adopted with probability less than $\frac{1}{n}$. With $d_{k}\left(x\right)=0$, referred to as random copying (50), all cultural variants are adopted with probability $\frac{1}{n}$. Finally, if $d_{k}\left(x\right)$ is negative (anticonformity), more widely dispersed variants ($g_{i,k}\left(x\right)<0$) are adopted with probability greater than $\frac{1}{n}$ and more tightly clustered variants ($g_{i,k}\left(x\right)>0$) are adopted with probability less than $\frac{1}{n}$.

Although $d_{k}\left(x\right)$ may depend on the observed opinions, x, as well as k, it is often chosen to be a constant $d_{k}\left(x\right)=d$ (50). To define $g_{i,k}\left(x\right)$, let the k-dispersal of variant $x_{i}$ be denoted by $f_{i,k}\left(x\right)$ (a non-negative number), and let the mean of the k-dispersals in a sample be $\bar{f_{k}}\left(x\right)=\sum_{i=1}^{n}f_{i,k}\left(x\right)/n$. Then, $g_{i,k}\left(x\right)$ is

[4] $$\begin{array}{cc} g_{i,k}\left(x\right) & =\left\{\begin{array}{c} -\frac{f_{i,k}\left(x\right)}{\sum_{z\in I}z}\;\text{if}\;f_{i,k}\left(x\right)\in I,\;I=\left\{z:z>\bar{f_{k}}\left(x\right)\right\}\\ \frac{{\left[f_{i,k}\left(x\right)\right]}^{-1}}{\sum_{z\in II}z^{-1}}\begin{array}{cc} & \text{if}\;f_{j,k}\left(x\right)>0\;\forall j\;\text{and}\;f_{i,k}\left(x\right)\in II,\\ & II=\{z:0<z<\bar{f_{k}}\left(x\right)\} \end{array}\\ \frac{1}{\sum_{j}1_{f_{j,k}\left(x\right)=0}}\;\text{if}\;f_{i,k}\left(x\right)=0\;\text{and}\;\bar{f_{k}}\left(x\right)>0\\ 0\;\text{if}\;\exists j,f_{j,k}\left(x\right)=0\;\text{and}\;0<f_{i,k}\left(x\right)<\bar{f_{k}}\left(x\right)\\ 0\;\text{if}\;f_{i,k}\left(x\right)=\bar{f_{k}}\left(x\right). \end{array}\right. \end{array}$$

As explained in ref. (50, p. 4], with equation numbers in the following quote updated to match the present paper: “If the dispersal of a variant $x_{i}$ is higher than the average dispersal of variants in the sample, i.e., $f_{i,k}\left(x\right)>\bar{f_{k}}\left(x\right)$, then it belongs to Group I (row 1 in Eq. **4**) and the value of $g_{i,k}\left(x\right)$ will be negative. In addition, $g_{i,k}\left(x\right)$ will become more negative as the dispersal $f_{i,k}\left(x\right)$ increases. Therefore, an anticonformist with $d_{k}\left(x\right)<0$ will have a higher probability of adopting a cultural variant as its dispersal increases (Eq. **3**). To understand row 2 in Eq. **4**, consider a case in which $f_{j,k}\left(x\right)>0$ for all j, and variant $x_{i}$ is less dispersed than the average dispersal in the sample, so $0<f_{i,k}\left(x\right)<\bar{f_{k}}\left(x\right)$. In this case, $g_{i,k}\left(x\right)$ will be positive, and will increase as the dispersal $f_{i,k}\left(x\right)$ decreases. Therefore, a conformist with $d_{k}\left(x\right)>0$ should have a higher probability of adopting a cultural variant $x_{i}$ as $x_{i}$’s dispersal from other variants decreases (Eq. **3**).” Rows 3 to 5 of Eq. **4** handle edge-cases and are discussed in further detail in ref. 50.

**Step 3:** error may be introduced into the adoption of opinions. For any opinion $x_{i}$ that the individual could choose from among the n observed opinions (in Step 2), $x_{i}$ is set as the mean of a normal distribution with SD σ. Then, because we have assumed that opinions must lie between 0 and 1, this normal distribution is truncated to [0,1]. If σ=0, there is no error and $x_{i}$ is copied exactly. If σ>0, the observer may adopt an opinion that deviates from $x_{i}$. The error term σ can also be introduced into the French–DeGroot models (see Section 1.4 below).

Note that as in ref. 50, after the normal distribution is truncated to [0,1], it may not have the same SD σ or mean $x_{i}$ as the original, nontruncated normal distribution. However, its new SD and mean tend to be close to σ and $x_{i}$, respectively, provided $x_{i}$ is not near 0 or 1. Here, as in ref. 50, σ and $x_{i}$ refer to the SD and mean of the normal distribution before truncation.

### 1.3. Extending the Conformity Model.

#### 1.3.1. Allowing n<3 individuals to be sampled.

The case of n<3 sampled individuals was not discussed in ref. 50, but it can be formulated by following the logic of their model. Step 1 in Section 1.2 is no longer needed because with only n=1 opinion, there is no notion of “dispersal” from other opinions, and with n=2 opinions, k must be 1 and the k-dispersals must be equal.

If n=1, Step 2 from Section 1.2 becomes deterministic: There is only one opinion that can be chosen. If n=2, then in Step 2, each opinion will have the same probability (50%) of being chosen—without k-dispersals that differ between opinions, there is no basis upon which to prefer one opinion over another.

Finally, Step 3 remains the same regardless of n: Each opinion that could have been chosen in Step 2 becomes the mean of a normal distribution with SD σ, truncated to [0,1].

#### 1.3.2. Including personal opinions.

In the model presented in Sections 1.2 and 1.3.1, an individual adopts an opinion solely based on the opinions of others. This model can be extended to incorporate individuals’ own, personal opinions as follows.

Let individual i’s personal opinion be denoted by $s_{i}$. The simplest way to incorporate this into the model is to expand the vector of observed opinions, $x=(x_{1},\cdots ,x_{n})$ to include $s_{i}$—namely, $x_{i}=\left(s_{i},x_{1},\cdots ,x_{n}\right)$—and proceed through Steps 1 to 3 in Section 1.2 as before.

However, it is possible that individuals place more (or less) weight on their own opinions than on the opinions of others. Let α be the weight placed on one’s own opinion relative to 1 for others, where α≥0.

The model is modified as follows. Step 2 in Section 1.2 produces a list of probabilities that an observer adopts opinion $x_{1},\cdots ,x_{n}$, denoted by $P_{k}\left(x_{1}|x\right),\cdots ,P_{k}\left(x_{n}|x\right)$, which must sum to 1. With $s_{i}$ included, there is one additional probability in this list, namely $P_{i,k}\left(s_{i}|x_{i}\right)$, where the vector x now includes $s_{i}$ and is therefore denoted by $x_{i}$ (and $P_{k}$ is now denoted by $P_{i,k}$). The other probabilities are also modified to depend on $x_{i}$ rather than x, namely $P_{i,k}\left(x_{1}|x_{i}\right),\cdots ,P_{i,k}\left(x_{n}|x_{i}\right)$. Then, to incorporate the weight placed on the observer’s own belief, we simply multiply $P_{i,k}\left(s_{i}|x_{i}\right)$ by α and multiply the other probabilities $P_{i,k}\left(x_{1}|x_{i}\right),\cdots ,P_{i,k}\left(x_{n}|x_{i}\right)$ by 1, and then renormalize so that all probabilities sum to 1. As there are n others’ opinions, this entails multiplying $P_{i,k}\left(s_{i}|x_{i}\right)$ by $\frac{\alpha }{\alpha +n}$ and the other probabilities by $\frac{1}{\alpha +n}$.

### 1.4. Making Comparisons Between Models.

Whereas the conformity model produces a probability density function over the possible opinions an individual may adopt, the one-stage and two-stage French–DeGroot models each produce a single value for the opinion that an individual will adopt. When comparing this predicted opinion to data, it will either be 100% correct—if a data point exactly matches the model prediction—or incorrect otherwise. In contrast, the conformity model can be more or less correct depending on the height of the model’s probability distribution that intersects with the real data. Because real data may be noisy, the French–DeGroot models might often be considered incorrect whereas the conformity model might often be considered “somewhat correct,” giving the conformity model an unfair advantage.

To make fair comparisons among the models, we need to either convert the conformity model’s output to a point estimate or determine the probability density function (PDF) of the French–DeGroot models. We believe the latter option is more interesting for two reasons. First, it is not always clear how one would convert the PDF from the conformity model to a single point estimate, especially if multiple peaks in the PDF are the same height. Second, we are interested in the model underlying opinion adoption instead of purely predicting revised opinions. A PDF captures more information about a model than a point estimate, allowing us to compare which model, French–DeGroot or conformity, is more probable given the observed revised opinions.

In the French–DeGroot model, we incorporate error (or uncertainty) in each individual’s estimate as follows. Suppose that individuals i=1,2,⋯,n have opinions $x_{1},x_{2},\cdots ,x_{n}$, respectively. From the perspective of a focal individual, let the normalized weights placed on these n opinions be $w_{1},w_{2},\cdots ,w_{n}$, with $0\leq w_{i}\leq 1$ for all i and $\sum_{i=1}^{n}w_{i}=1$. These weights may be obtained from either the trust matrix, $T^{\left(t\right)}$, or the squared trust matrix, ${\left(T^{\left(t\right)}\right)}^{2}$ (Eqs. **1** or **2**), for the one- or two-stage French–DeGroot model, respectively. Then, the weighted average guess is

$$\begin{array}{cc} \bar{x} & =\sum_{i=1}^{n}w_{i}x_{i}. \end{array}$$

If each individual’s opinion were formed independently, prior to learning about others’ opinions (as we will assume throughout this paper), then the SD of $\bar{x}$ is

[5] $$\begin{array}{cc} \sigma_{\bar{x}} & =\sigma \sqrt{\sum_{i=1}^{n}w_{i}^{2}}. \end{array}$$

**Fig. 1.**
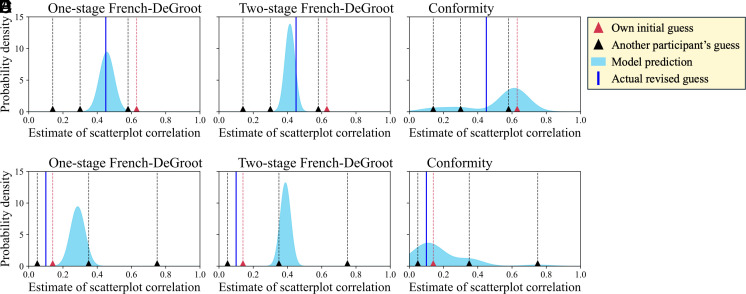
Two examples from the dataset in ref. 37. In panels (*A*–*C*), an individual’s initial estimate of a scatter plot correlation was 0.63 (red triangle); this individual observed n=3 others’ initial estimates to be 0.14, 0.3, and 0.58 (black triangles), and gave a revised estimate of 0.45 (blue line) (this example is given in Table 1). In panels (*D*–*F*), an individual’s initial estimate of a scatter plot correlation was 0.14 (red triangle); this individual observed n=3 others’ initial estimates to be 0.05, 0.35, and 0.75 (black triangles), and gave a revised estimate of 0.1 (blue line). The light blue probability distributions in panels (*A* and *D*) are normal distributions with SD given by Eq. **5** with σ=0.08, centered around the predicted estimate from the one-stage French–DeGroot model. The parameter α is set to 2 (i.e., individuals place twice as much weight on their own opinions as on the opinions of others). In panels (*B* and *E*), the parameter values are the same and the normal distribution is centered on the predicted estimate from the two-stage French–DeGroot model. In panels (*C* and *F*), the probability density curve is obtained from a conformity model similar to ref. 50 with their error parameter σ=0.08, k=2, and conformity parameter d=0.8, but revised to include personal opinions with weight α=2. In the *Top* row, the one-stage French–DeGroot model best fits the data as the dark blue line (real behavior) intersects the light blue probability density function (model behavior) at a higher point, $y^{*}\approx 9.3$, than in the other models [$y^{*}\approx 6.5$ in panel (*B*), and $y^{*}\approx 0.7$ in panel (*C*)]. In contrast, in the *Bottom* row, the conformity model provides the best fit to the data [$y^{*}\approx 3.6$ in panel (*F*), in contrast to $y^{*}<0.0007$ in panels (*D* and *E*)].

Therefore, we set the predicted value from the one- or two-stage French–DeGroot model (Eqs. **1** or **2**, respectively) as the mean of a normal distribution with SD $\sigma_{\bar{x}}$. As the opinions in the data are restricted to [0,1] (37), this normal distribution is then truncated to that domain. Fig. 1 (discussed below in Section 2.2) shows examples of all three models with k=2, d=0.8, α=2, and σ=0.08. Later, when applying the models to the data, we will consider two cases for the conformity model: first, we will find the best-fitting σ and α parameters—denoted by $\hat{\sigma }$ and $\hat{\alpha }$, respectively—while keeping k and d fixed to enable a fair comparison between models with two free parameters. Second, we will consider the full conformity model with all of its free parameters.

Finally, note that when α→∞, the conformity model, one-stage French–DeGroot model, and two-stage French–DeGroot model become similar. In this case, individuals consider only their own, initial opinions and disregard the opinions of others. Then, their initial opinions are set as the mean of a normal distribution with SD σ (for the conformity model) or $\sigma_{\bar{x}}$ (for the French–DeGroot models).

## 2. Evaluating Models on Data

### 2.1. Description of the Dataset.

Almaatouq et al. (37) conducted two online experiments, referred to as $E_{1}$ and $E_{2}$, in which $N_{E_{1}}=719$ and $N_{E_{2}}=702$ participants completed 20 estimation tasks (i.e., “rounds” of a game). In each round, participants were asked to estimate the correlation between 0 and 1 of points in a scatter plot.

Each participant was assigned to a group of 12 and an experimental condition (together referred to as “a game”), which remained the same for the duration of the 20 rounds. In experiment $E_{1}$, the presence of feedback was held constant: After each round of the experiment, individuals received feedback on the correct correlation and how accurate their guess was, as well as how accurate others’ guesses were that they had viewed (if present). There were three experimental conditions, namely

(i) $E_{1}$ Solo: participants could not view the answers of other participants.
(ii) $E_{1}$ Static: participants viewed the answers of a fixed set of three other individuals for all rounds of the experiment.
(iii) $E_{1}$ Dynamic: in each round of the experiment, participants could choose to view the answers of up to three individuals (who could be different at each round).

Experiment $E_{2}$ contained four conditions:

(iv) $E_{2}$ Solo: participants could not view the answers of other participants or receive feedback about how accurate their own guesses were.
(v) $E_{2}$ No Feedback: participants belonged to a dynamic network [similar to (iii) above] but did not receive performance feedback about their own or others’ guesses.
(vi) $E_{2}$ Self Feedback: participants belonged to a dynamic network and received performance feedback about their own guess, but not others’ guesses.
(vii) $E_{2}$ Full Feedback: participants belonged to a dynamic network and received performance feedback about both their own guess and others’ guesses.

In each round of the experiment, all participants were shown scatter plots that shared the same true correlation. However, the number of points, outliers, and nonlinearities in the data could vary, making the task easier or more difficult for different participants (referred to as the “signal quality”) (37). Specifically, there were three kinds of signal quality: low (most difficult), medium, and high (least difficult). In the first ten rounds of the experiment, a participant was shown scatter plots of the same signal quality, whereas at round 11, the signal quality was changed, and remained at the new quality for all subsequent rounds.

Under the “nonsolo” conditions in which participants viewed others’ answers, namely conditions (ii), (iii), and (v)–(vii), individuals could see each others’ initial answers and subsequent answers as they were updated in real time (37). Here, we are interested in how others’ initial answers, based solely on personal beliefs, affect a focal individual’s decision, rather than asking how others’ updated answers affect the focal individual’s decision. The reason is that it is difficult to isolate the effects of one’s own, personal opinion (with weight α) and others’ opinions if the others’ opinions change in response to that personal opinion and vice versa. Similarly, in the model of ref. 37, agents update their beliefs based on others’ personal opinions, or “private signals,” rather than based on others’ revised opinions following social exposure.

### 2.2. Data Analysis.

Table 1 provides an example of a single row of the dataset. Fig. 1 illustrates two examples in which the one-stage French–DeGroot, two-stage French–DeGroot, and conformity models are compared to the data from ref. 37; the *Top* row of Fig. 1 includes the data from Table 1 and the *Bottom* row of Fig. 1 includes a different row of the data. Thus, in each row of Fig. 1, only one round of the experiment is shown for one focal individual, and the same data are represented across all three columns in that row so that the match between the three models and the data can be compared.

**Table 1.** Example of a single row in the dataset^∗^

Condition	Game ID	Round number	Correct answer	Player ID	Player’s initial guess	Person 1’s initial guess	Person 2’s initial guess	Person 3’s initial guess	Player’s revised guess
*E*_1_ Static	86	1	0.09	d6f9...	0.63	0.58	0.14	0.3	0.45

^∗^For each condition ($E_{1}$ Static and Dynamic; $E_{2}$ No Feedback, Self Feedback, and Full Feedback), and for each game ID, round number, and player ID, there is a row in the dataset of ref. 37.

In the *Top* row of Fig. 1, the one-stage French–DeGroot model in panel (*A*) matches the data most closely because the light blue curve (predicted revised guess) intersects the dark blue line (actual revised guess) at a higher point than in panels (*B* or *C*). Specifically, if we denote the height at which the probability curve intersects the actual revised guess by $y^{*}$, then $y^{*}\approx 9.3$ in panel (*A*), $y^{*}\approx 6.5$ in panel (*B*), and $y^{*}\approx 0.7$ in panel (*C*). In the *Bottom* row of Fig. 1*D*–*F*, the conformity model matches the data more closely than either of the French–DeGroot models. Here, the light blue curve in panel (*F*) intersects the dark blue line at a higher point than in panels (*D* and *E*) [specifically, $y^{*}<0.0007$ in panels (*D* and *E*) whereas $y^{*}\approx 3.6$ in panel (*F*)].

Fig. 1 shows model predictions for one set of parameter values in two rows of a dataset that contains thousands of rows (37). We repeat the process of finding $y^{*}$ for (*A*) the one-stage French–DeGroot model, (*B*) the two-stage French–DeGroot model, and (*C*) the conformity model for all rows of the dataset and across a variety of parameter values. Then, we use this information to answer the following questions.

**Questions:** For the conditions in the experiment of ref. 37 in which individuals viewed others’ answers (i.e., all nonsolo conditions):

1. Within each model (one-stage French–DeGroot, two-stage French–DeGroot, and conformity), which parameter values, such as σ and α, provide the best fit to the data? These best-fitting parameters are marked with a ^ symbol (e.g., $\hat{\sigma }$ and $\hat{\alpha }$, respectively).
2. Of the best-fitting one-stage French–DeGroot, two-stage French–DeGroot, and conformity models, which provides the best fit to the data?
3. Do the answers to Questions 1 and 2 change across different experimental conditions in ref. 37 ($E_{1}$ Static and Dynamic; $E_{2}$ No Feedback, Self Feedback, and Full Feedback)?

To address Question 1, we assessed the fit between the data and a given model, with various parameter values explored using an optimization algorithm (discussed below and in *SI Appendix*, section 2). We examined two versions of the conformity model (*SI Appendix*, Table S1). In the “two-parameter” conformity model, α and σ were the only free parameters (as in the French–DeGroot models) while d was fixed at 0.8 and k was fixed at 2, as in several of the simulations in ref. 50 (see their figures 4–7). In the “four-parameter” conformity model, α, σ, d, and k all were free parameters.

To avoid overfitting the data, we explored the various parameter values on only 50% of the data (referred to as the “training data”), which was distinct from the other 50% of the data used to answer Question 2 (the “test data”). Specifically, because the dataset included 20 rounds of an experiment, we extracted the odd-numbered rounds (1,3,5,⋯,19) for the training data used for parameter estimation, and the even-numbered rounds for the test data used for comparing different models’ performance to one another.

Altogether, the dataset from ref. 37 contained 14,230 rows with odd-numbered rounds and 14,230 with even-numbered rounds. After removing the “solo” conditions in which individuals did not view others’ guesses, there were 10,200 rows with odd-numbered rounds and 10,200 rows with even-numbered rounds. Finally, once we removed rows in which an individual did not submit an independent guess or a revised guess, the number of rows with odd-numbered rounds was 9,931 and the number of rows with even-numbered rounds was 9,971.

On the training data (9,931 rows), we searched for the best-fitting parameters using hyperparameter optimization via Python’s HyperOpt library (55). Specifically, within HyperOpt we selected the Bayesian optimization algorithm TPE (56) (Tree-structured Parzen Estimator; see *SI Appendix*, section 2). Compared to other methods for parameter optimization, such as gradient-based methods, HyperOpt with TPE is better suited for optimizing functions that are irregular or whose analytical form is not known. We sought to optimize three kinds of functions, described below.

Recall that for a given combination of parameter values in a given model (e.g., conformity or French–DeGroot), we measured how well the model fit the data by calculating the height $y^{*}$ of the probability density function curves that intersected with individuals’ revised guesses (as in each row of Fig. 1). The higher the $y^{*}$ values, the better the fit between the model and the data. We summarized the $y^{*}$ values in a statistic, denoted by ${\tilde{y}}^{*}$, which was set as the output of a function that our algorithm (HyperOpt with TPE) sought to maximize. Specifically, we defined the summary statistic ${\tilde{y}}^{*}$ as the sum of natural logs of the $y^{*}$ values, denoted by ${\tilde{y}}_{\text{sumlogs}}^{*}$. We also explored the results when ${\tilde{y}}^{*}$ was defined as the mean of the $y^{*}$ values (${\tilde{y}}_{\text{mean}}^{*}$) and the median of the $y^{*}$ values (${\tilde{y}}_{\text{median}}^{*}$) in both *SI Appendix*, sections 2 and 3. Of these three options for the summary statistic, ${\tilde{y}}_{\text{sumlogs}}^{*}$ is the most standard, with several advantages (*Discussion*).

To interpret different values of these summary statistics, consider the null example of a probability density function that is uniform on [0, 1]. In this case, all values of $y^{*}$ will be 1, so ${\tilde{y}}_{\text{sumlogs}}^{*}=0$, ${\tilde{y}}_{\text{mean}}^{*}=1$, and ${\tilde{y}}_{\text{median}}^{*}=1$. Higher values of these summary statistics indicate that the model provides a better fit to the data than the uniform distribution. For instance, if a model consistently produces $y^{*}=\phi$ (i.e., it is ϕ times as predictive of the data as the uniform distribution), then ${\tilde{y}}_{\text{mean}}^{*}=\phi$, ${\tilde{y}}_{\text{median}}^{*}=\phi$, and if there are ℓ data points, ${\tilde{y}}_{\text{sumlogs}}^{*}=\ell \log\left(\phi \right)$. Values of ℓ are given in *SI Appendix*, Table S2.

For a given French–DeGroot or conformity model, the parameter values that are found (via HyperOpt with TPE) to maximize the summary statistic, ${\tilde{y}}^{*}$, on the training data are considered its “best-fitting” parameter values. On the test data, we compared each model with its best-fitting parameter values to the null, uniform distribution (*SI Appendix*, section 2) and to the other models (*SI Appendix*, section 3, addressing Question 2).

Specifically, we compared the $y^{*}$ values of two models at a time (e.g., one-stage French–DeGroot vs. two-parameter conformity, or one-stage French–DeGroot vs. the null distribution), as opposed to multiple models simultaneously, using the pairwise t test and the Wilcoxon signed-rank test—both two-tailed. To account for the fact that each participant played multiple rounds of the game (corresponding to multiple $y^{*}$ values), which constitute dependent observations, we averaged each participant’s $y^{*}$ values prior to running these statistical tests. Using the Shapiro–Wilk test of normality, we determined that pairwise differences between models in these per-participant averaged $y^{*}$ values were often not normally distributed, and therefore some would argue that the nonparametric Wilcoxon signed-rank test is more appropriate than the pairwise t test for analyzing the data. However, others have contended that “the t-test is so robust against non-normality that there is nearly no need to use the Wilcoxon test” (57, p. 175]. For completeness, we report the results of both analyses.

Finally, to address Question 3, we conducted all of the above analyses on the five different nonsolo conditions represented in the data ($E_{1}$ Static, $E_{1}$ Dynamic, $E_{2}$ No Feedback, $E_{2}$ Self Feedback, and $E_{2}$ Full Feedback).

### 2.3. Results of Data Analysis.

The results, reported in *SI Appendix*, Tables S3–S20, include five experimental conditions ($E_{1}$ Static and Dynamic; $E_{2}$ No Feedback, Self Feedback, and Full Feedback) and three summary statistics used to obtain the best-fitting parameters (${\tilde{y}}_{\text{sumlogs}}^{*}$, ${\tilde{y}}_{\text{mean}}^{*}$, and ${\tilde{y}}_{\text{median}}^{*}$). Below, we will focus primarily on the results for ${\tilde{y}}_{\text{sumlogs}}^{*}$ and provide a brief overview of the results for ${\tilde{y}}_{\text{mean}}^{*}$ and ${\tilde{y}}_{\text{median}}^{*}$, with further discussion in both *SI Appendix*, sections 2.B and 3.B.

#### 2.3.1. Best-fitting parameter values.

When the summary statistic was ${\tilde{y}}_{\text{sumlogs}}^{*}$, the best-fitting error parameters $\hat{\sigma }$ were lower for the two- and four-parameter conformity models ($\hat{\sigma }<0.08$) than for the one- and two-stage French–DeGroot models ($\hat{\sigma }>0.2$) (*SI Appendix*, Table S3). Values of $\hat{\alpha }$ ranged from about 3.1 to 8.6 for the conformity models and from about 2.8 to 14.8 for the French–DeGroot models depending on the experimental condition (*SI Appendix*, Table S3). Notably, in each conformity and French–DeGroot model, $\hat{\alpha }$ values were lower for the experimental conditions $E_{1}$ Dynamic and $E_{2}$ Full Feedback than for the other three conditions. $E_{1}$ Dynamic and $E_{2}$ Full Feedback are the only two experimental conditions in which participants can a) choose their “neighbors,” i.e., choose whose answers to view, rather than being assigned neighbors, and b) receive feedback on the accuracy of their own and their neighbors’ estimates. It makes sense that when given a choice of neighbors and information about their accuracy, participants may have higher confidence in their neighbors’ estimates and decrease the relative weight placed on their own estimates, $\hat{\alpha }$, than when neighbors are assigned arbitrarily or when their estimation accuracies are unknown.

In addition to σ and α, the four-parameter conformity model included d and k. Best-fitting values of $\hat{d}$ were positive for all five experimental conditions (*SI Appendix*, Table S3), indicating that individuals exhibited conformity rather than anticonformity (if $\hat{d}<0$) or random copying (if $\hat{d}=0$). Best-fitting values of $\hat{k}$ were either 1 or 2 (*SI Appendix*, Table S3). Recall that the $\hat{k}$ parameter determines, for a given cultural variant, how many other cultural variants must be close to it for it to be considered “popular” (Section 1.2). For example, if four individuals estimated a scatter plot correlation to be 0.091, 0.1, 0.99, 1, then with k=1, the guess 0.091 may be considered popular (i.e., near one other guess, not “standing out” alone; see also ref. 58), but with k=2 it would not be considered popular.

So far, we have discussed the best-fitting parameter values for the case in which the summary statistic was ${\tilde{y}}_{\text{sumlogs}}^{*}$. When the summary statistic was instead ${\tilde{y}}_{\text{mean}}^{*}$ or ${\tilde{y}}_{\text{median}}^{*}$, the results differed; in particular, $\hat{\alpha }$ values were much larger (*SI Appendix*, Tables S4 and S5). In the four-parameter conformity model with large $\hat{\alpha }$ (i.e., when individuals placed a large weight on their own opinions relative to the opinions of others), the parameter $\hat{d}$ was negative, indicating anticonformity rather than conformity to others’ opinions (*SI Appendix*, Tables S4 and S5), whereas for the few cases in which $\hat{\alpha }$ was low and others’ opinions were weighted more heavily, there was conformity with $\hat{d}>0$ (*SI Appendix*, Table S5). To explore potential factors that may have driven most $\hat{\alpha }$ values to be large in the conformity and French–DeGroot models, we analyzed two subsets of the data: in subset A, we omitted instances in which individuals did not revise their guesses, and in subset B, we omitted players who tended to revise their guesses by only a small amount across the 20 rounds of the game (defined in *SI Appendix*, Table S1 and Section 2.B). We found that with ${\tilde{y}}_{\text{mean}}^{*}$, high $\hat{\alpha }$ values appeared to be primarily driven by cases in which individuals’ revised guesses were identical to their initial guesses, whereas with ${\tilde{y}}_{\text{median}}^{*}$, both cases of zero-revision and low-revision players contributed to high $\hat{\alpha }$ values (discussed further in *SI Appendix*, section 2.B).

#### 2.3.2. Comparing the best-fitting models to the null distribution.

With ${\tilde{y}}_{\text{sumlogs}}^{*}$, all of the French–DeGroot and conformity models, with their best-fitting parameters, provided a better fit to the test data than the null (uniform) distribution when analyzing the data in full, in subset A, and in subset B. The same was true for ${\tilde{y}}_{\text{median}}^{*}$

in all three data types, and for ${\tilde{y}}_{\text{mean}}^{*}$ in the full data. For ${\tilde{y}}_{\text{mean}}^{*}$, the French–DeGroot models sometimes provided a poorer fit to the subset data than the null model (e.g., *SI Appendix*, Table S7, and see discussion in *SI Appendix*, section 2.B).

#### 2.3.3. Comparing the best-fitting models to each other.

All statistical analyses and associated P-values are reported in *SI Appendix*, Tables S12–S20. For ${\tilde{y}}_{\text{sumlogs}}^{*}$ in all three data types (full, subset A, and subset B), the conformity models provided a significantly better fit to the data than the French–DeGroot models across all experimental conditions. The same was almost always true for ${\tilde{y}}_{\text{mean}}^{*}$ (but see one exception in *SI Appendix*, Table S19 where the P-value did not indicate significance). For ${\tilde{y}}_{\text{median}}^{*}$, the results were less straightforward in the full data and subset A data, discussed in detail in *SI Appendix*, section 3.B, but in subset B, the conformity models provided a significantly better fit to the data than the French–DeGroot models.

Overall, it appears that in several cases, the conformity model more closely corresponds to individuals’ choices in the experimental study of ref. 37 than the French–DeGroot models. Our next question is whether the outcomes of theoretical analyses that invoke French–DeGroot updating would differ if conformity were implemented instead. If French–DeGroot updating and conformity produced similar hypothetical population-level outcomes, then even if one model matched empirical data more closely, the predominantly theoretical literature that has invoked French–DeGroot updating would not be affected. If the conformity model produced different population-level predictions from the French–DeGroot models, it may be interesting to reanalyze previous work that employed French–DeGroot updating with conformity implemented instead to determine whether, and how, the conclusions would change.

## 3. Evolutionary Simulations

Here, we present six simple simulations showing some of the possible population-level consequences of French–DeGroot updating and conformity. It is important to note that, as in ref. 37, our simulations are not aimed at replicating the empirical data. We explore larger populations of interacting individuals over much longer time-scales than in the experiment to evaluate the long-term evolutionary consequences of each model.

Our simulations explore a) different types of population structure and b) different initial distributions of opinions. With respect to a), we consider the following three populations. Population 1 is a fully connected (complete) network, equivalent to a single well-mixed population as in ref. 50. Population 2 is a static network, namely a directed Erdős-Rényi-Gilbert graph (59, 60). Population 3 is an adaptive network in which individuals preferentially connect to individuals with more accurate estimates, similar but not identical to the simulations of ref. 37. Here, the initial network at time t=0 is identical to Population 2.

With respect to b) the initial distributions of opinions, we constructed two scenarios. First, the initial opinion of each individual in the population was drawn from a uniform distribution on [0,1]. Second, half of the population’s initial opinions were drawn from a uniform distribution on [0,0.2] and the other half of the population’s initial opinions were drawn from a uniform distribution on [0.8,1], referred to as a highly polarized population. Polarization can be quantified using the index F introduced in ref. 61 (and used in ref. 50): if x is the vector of opinions in a population, then the polarization index $F=\frac{\text{Var}(x)}{\text{Mean}(x)\cdot [1-\;\text{Mean}(x)]}$. F=0 indicates no polarization whereas F=1 indicates maximum polarization. For each simulation, in addition to reporting the average opinions over time, we also report the polarization indices over time in *SI Appendix*, section 4.

In all simulations, we assumed that the population comprised N=100 individuals and each individual sampled the opinions of up to n=3 others (although this number could be smaller if the individual was not connected to n=3 others). The parameter values were σ=0.08, α=2, d=0.8, and k=2 as in Fig. 1. We describe further details about the populations and their dynamics below.

### 3.1. Population 1.

**Fig. 2.**
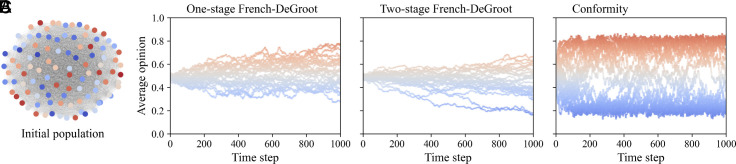
Evolutionary simulation results for the simplest scenario of N=100 individuals in a single complete network. Each individual’s initial opinion is drawn from a uniform distribution on [0,1]. More pronounced blue colors correspond to opinions closer to 0 and more pronounced red colors correspond to opinions closer to 1. Panels (*A*–*C*) show the one-stage French–DeGroot model, two-stage French–DeGroot model, and conformity model, respectively. Each plot includes 30 replicates of the model and shows the average opinion in the population for each of these replicates over 1,000 time steps. The average opinion is colored more blue as it approaches 0 and more red as it approaches 1. The parameters of the model are n=3, σ=0.08, α=2, d=0.8, and k=2, as in Fig. 1.

In the simplest model, there is one large group of N individuals with initial opinions sampled uniformly on [0,1] (Fig. 2). Each individual takes a random sample of n=3 others in the population and applies their updating rule (either one-stage French–DeGroot, two-stage French–DeGroot, or conformity). This process is repeated for 1,000 time steps, with individuals’ personal opinions at time t+1 set to their revised opinions at time t. Fig. 2 shows 30 replicates of the simulation for each of the three models, and reveals that compared to the French–DeGroot models, the conformity model often causes the population’s average opinion to be considerably higher or lower than 0.5 in fewer time steps. In the case where half of the initial opinions are sampled uniformly on [0,0.2] and the other half are sampled uniformly on [0.8,1], the results are similar (*SI Appendix*, Fig. S1). *SI Appendix*, Figs. S2 and S3 show the polarization indices, F, over time for the plots in Fig. 2 and *SI Appendix*, Fig. S1, respectively. Whereas populations rapidly reach low polarization (F close to 0) in the French–DeGroot models, F values remain higher with conformity.

### 3.2. Population 2.

The initial static network is a directed Erdős-Rényi-Gilbert network. Each individual in the population has a probability, which we set to be 0.2, of forming a connection (i.e., a directed edge) to any other individual. The term “directed” means that individual A may connect to individual B (A→B) even if individual B does not connect to individual A. If A is connected to B, then A may sample the opinion of B—although individuals sample the opinions of n of their connections at each time step and may or may not observe all of their connections’ opinions over the course of the simulation. The results are similar to those for Population 1 and are given in *SI Appendix*, Figs. S4 and S5, with polarization indices in *SI Appendix*, Figs. S6 and S7.

### 3.3. Population 3.

In addition to the static network explored in Population 2 (with parallels to the experimental condition $E_{1}$ Static), in Population 3 individuals can adaptively rewire their connections based on feedback about others’ performance (with parallels to $E_{1}$ Dynamic and $E_{2}$ Full Feedback, and similar to the numerical simulations of ref. 37). Initially, Population 3 is identical to Population 2, but over the course of the simulation individuals can form different connections. Here, we introduce the concept of “correctness”: Some opinions are closer to the truth than others. Specifically, we set the truth to be ω=0.9 (e.g., if opinions are about a scatter plot correlation, the true correlation is 0.9). As in the simulations of ref. 37, the correct opinion ω is different from the mean of the initial distribution of opinions in the population—which, here, we expect to be 0.5 as the initial opinions are either sampled from a uniform distribution on [0,1], or half of the opinions are sampled from a uniform distribution on [0,0.2] and the other half are sampled from a uniform distribution on [0.8,1].

As in the simulations of ref. 37, we assume that individuals do not know the truth, ω, but receive information about the absolute error in their own and others’ estimates after each time step. Then, they can choose to form new network connections depending on these errors (e.g., detach from a neighbor with high error). However, unlike in ref. 37, we do not assume that individuals have complete information about the errors of all other individuals in the population. Instead, we assume that individuals only know the errors of their current neighbors and n=3 randomly sampled others.

Finally, as in the simulations for Populations 1 and 2, we distinguish between individuals’ initial estimates based on personal beliefs and revised estimates based on social information. However, here, we set individuals’ personal beliefs to be their initial opinions, and keep these values the same for the duration of the simulation rather than updating them in each time step. This way, some individuals are intrinsically more or less correct in their beliefs about the truth (ω), similar to the model of ref. 37.

The simulation proceeds as follows. i) The initial network is constructed and initial opinions are obtained, as described previously. ii) Individuals revise their opinions following either the one-stage French–DeGroot, two-stage French–DeGroot, or conformity model, depending on the condition. The revised opinions of all individuals in the population are denoted by $x_{1},x_{2},\cdots ,x_{N}$. iii) Individuals receive information about the absolute error $\epsilon_{i}=\left|x_{i}-\omega \right|$ of their own revised opinion as well as their neighbors’ revised opinions. iv) Each individual i detaches from any of its neighbors j whose absolute error $\epsilon_{j}$ is higher than its own ($\epsilon_{j}>\epsilon_{i}$). v) Each individual i samples n=3 other individuals in the population (non-neighbors) and attaches to any of these 3 individuals whose absolute error $\epsilon_{j}$ is lower than its own (i.e., $\epsilon_{j}<\epsilon_{i}$).

**Fig. 3.**
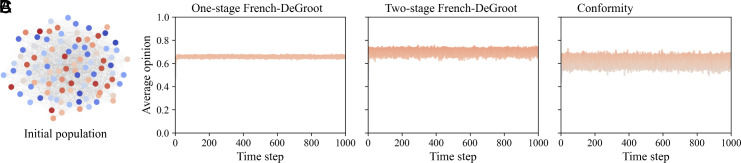
Evolutionary simulation results for the adaptive network model (“Population 3” described in Section 3). N=100 individuals are initially in a network shown on the left-hand side, where each individual’s initial opinion is drawn from a uniform distribution on [0,1]. More pronounced blue colors correspond to opinions closer to 0 and more pronounced red colors correspond to opinions closer to 1. Panels (*A*–*C*) show the dynamics under the one-stage French–DeGroot model, two-stage French–DeGroot model, and conformity model, respectively. Each panel includes 30 replicates of the model and shows the average opinion in the population for each of these replicates for 1,000 time steps. The parameters of the model are n=3, σ=0.08, α=2, d=0.8, and k=2, as in Fig. 1, and ω=0.9 is set as the correct opinion.

The results of this simulation are illustrated in Fig. 3 and *SI Appendix*, Fig. S8, with polarization indices in *SI Appendix*, Figs. S9 and S10, respectively. In all models (one-stage French–DeGroot, two-stage French–DeGroot, and conformity), the average opinion in the population fell below the true value of ω=0.9. This makes sense, as the average of all individuals’ personal opinions was either 0.486 (Fig. 3) or 0.492 (*SI Appendix*, Fig. S8), and individuals placed a certain weight, α=2, on their personal opinions throughout the simulation. This prevented them from achieving perfect “wisdom of the crowd,” i.e., converging on ω=0.9.

Interestingly, compared to the French–DeGroot models, the conformity model produced average opinions that were farther from the truth ω=0.9, thus decreasing the wisdom of the crowd (Fig. 3 and *SI Appendix*, Fig. S8). Although the exact mechanism by which conformity reduces crowd wisdom relative to the French–DeGroot models is not clear, one notable difference between the conformity and French–DeGroot models is in the extent to which they sustained population-level polarization over time. *SI Appendix*, Figs. S9 and S10 show that the conformity model produced significantly higher levels of polarization than either of the French–DeGroot models in the simulations of Fig. 3 and *SI Appendix*, Fig. S8, respectively. Future research could explore whether there is a causal link between increasing polarization and decreasing crowd wisdom, or vice versa, in these and other models.

## 4. Discussion

In this study, we investigated two mechanisms by which individuals may acquire opinions. One was averaging the opinions of others, as in the widely used French–DeGroot model (38, 39). The other was conformity to clusters of similar opinions among others (50). In the conformity model, individuals sample from a probability distribution around individuals’ opinions with peaks that depend on how similar (i.e., closely clustered) they are to each other.

This model of conformity is intuitive, particularly when considering cases in which the average of several opinions is not similar to any one individual’s opinion. For example, suppose four individuals estimate the percent of red jelly beans in a jar, and two guess 100% while two guess 0%. An observer might infer that all of the jelly beans in the jar are the same color, but that some individuals are interpreting this color as red (and guessing 100%) while others are interpreting this color as, say, orange (and guessing 0%). Therefore, the observer might guess either 100% or 0%—consistent with the conformity model—as opposed to averaging others’ guesses and estimating that 50% of the jelly beans are red and 50% are a different color. In another example, suppose that three individuals estimated the correlation of points in a scatter plot to be 1 and one individual guessed that the correlation was 0. An observer might infer that there is a strong positive correlation in the scatter plot but that one individual misunderstood the question, and submit a guess of 1 rather than the average (0.75).

In addition to others’ opinions, individuals’ own, personal opinions often affect their choices. For example, suppose that an individual estimated the percent of red jelly beans in a jar to be 10%. If a small number of others disagreed, this individual may nevertheless adhere to the guess of 10%. On the other hand, if a large number of others made a different estimate (say, 30%), the individual may begin to doubt the initial guess of 10% and align with the more popular guess. This phenomenon has been widely observed in Aschian line judgment tasks, where individuals estimate the relative lengths of different lines and may abandon the clearly correct answer (e.g., “line A is shortest”) in favor of a majority’s incorrect answer (52–54). Thus, it is likely that people place a certain weight on their own opinions relative to those of others and may be swayed away from their original guesses if this weight is not too large and if enough other people disagree with them. In this study, we denoted the weight that individuals placed on their own guesses by α, while the weight they placed on any other individual’s guess was 1. As the number of individuals n observed becomes large, the absolute weight placed on one’s own guess, $\frac{\alpha }{\alpha +n}$, decreases, in line with the expectations of empirical studies on Aschian line judgment tasks.

The weight α was one of the parameters in the conformity, one-stage French–DeGroot, and two-stage French–DeGroot models that we estimated using experimental data. Another parameter was the error term, σ. In the conformity model, σ determines whether individuals adopt the exact opinion of one of the individuals they observe (σ=0) or whether their opinion can deviate from these opinions (σ>0). Although French–DeGroot models do not typically include an error term, this parameter can be introduced (Section 1.4), in which case their outputs become probability distributions—as in the conformity model—rather than point estimates. This enables a fair comparison between the conformity and French–DeGroot models when tested on data. In addition to α and σ, two other parameters in the conformity model are d, which controls the extent of conformity (if it is positive) or anticonformity (if it is negative), and k, a dispersal parameter defined in Section 1.2. These parameters do not appear in the French–DeGroot models. In the “two-parameter” conformity model, we fixed d=0.8 and k=2 so that the number of free parameters was the same as in the French–DeGroot models, whereas in the “four-parameter” conformity model we allowed all of the parameters to be inferred from the data.

The data that we used were from an experiment conducted by Almaatouq et al. (37). In this experiment, individuals made an initial estimate of a scatter plot correlation (between 0 and 1), viewed the estimates of up to three other individuals, and decided whether to revise their guesses or stick with their original guesses. Participants were assigned to one of seven experimental conditions, including two control conditions in which they did not view others’ estimates. Here, we focused on the five conditions in which participants could view others’ estimates, labeled $E_{1}$ Static, $E_{1}$ Dynamic, $E_{2}$ No Feedback, $E_{2}$ Self Feedback, and $E_{2}$ Full Feedback (Section 2.1). We explored these data in full, as well as two subsets of the data denoted by A and B. Subset A excluded cases in which individuals’ revised guesses were identical to their initial guesses, enabling us to focus on cases in which opinions changed. Subset B excluded participants who tended not to revise their guesses very much over the 20 rounds of the game, defined in *SI Appendix*, Table S1.

We obtained the best-fitting parameter values for each model on the training data (50% of the data) using a Bayesian optimization algorithm (55, 56) described in *SI Appendix*, section 2. This algorithm maximized a statistic, denoted by ${\tilde{y}}^{*}$, that summarized many $y^{*}$ values produced by a model. A $y^{*}$ value is the point on the y-axis of a plot (height) at which a model’s probability density function—i.e., prediction of an individual’s revised guess—intersects with the individual’s actual revised guess; see Fig. 1 for examples. Higher $y^{*}$ values indicate a better fit between a model and the data.

There are several ways to summarize many $y^{*}$ values in a single statistic, ${\tilde{y}}^{*}$. For example, ${\tilde{y}}^{*}$ could be the mean of the $y^{*}$ values, denoted by ${\tilde{y}}_{\text{mean}}^{*}$. However, the mean can be heavily influenced by outliers; e.g., a small number of extremely large peaks in a model’s probability density function that intersect with an individual’s revised guess and produce large $y^{*}$ values can inflate ${\tilde{y}}_{\text{mean}}^{*}$, even if the majority of other $y^{*}$ values are very low. Alternatively, ${\tilde{y}}^{*}$ could be the median of the $y^{*}$ values, denoted by ${\tilde{y}}_{\text{median}}^{*}$. The median represents the typical $y^{*}$ value, but does not capture information about how small the smallest $y^{*}$ values are or how large the largest $y^{*}$ values are. Thus, a disadvantage of using ${\tilde{y}}_{\text{median}}^{*}$ over ${\tilde{y}}_{\text{mean}}^{*}$ is that the median is not affected by the magnitudes of all the $y^{*}$ values.

A third option for the summary statistic, ${\tilde{y}}^{*}$, is the sum of natural logs of the $y^{*}$ values, denoted by ${\tilde{y}}_{\text{sumlogs}}^{*}$. Like ${\tilde{y}}_{\text{mean}}^{*}$, ${\tilde{y}}_{\text{sumlogs}}^{*}$ takes into account all of the data points, providing an advantage over ${\tilde{y}}_{\text{median}}^{*}$. In addition, compared to ${\tilde{y}}_{\text{mean}}^{*}$, ${\tilde{y}}_{\text{sumlogs}}^{*}$ is less affected by extremely large $y^{*}$ values; their magnitudes are reduced when the log is applied. On the other hand, very small $y^{*}$ values, approaching zero, can greatly affect the value of ${\tilde{y}}_{\text{sumlogs}}^{*}$, as $y^{*}\to 0$ causes $\log(y^{*})\to -\infty$. Therefore, the model that maximizes ${\tilde{y}}_{\text{sumlogs}}^{*}$ tends to provide a reasonable fit to all data points, with few $y^{*}$ values near 0. In considering the advantages and disadvantages of these three summary statistics, we viewed ${\tilde{y}}_{\text{sumlogs}}^{*}$ as the best suited for our analyses and focused primarily on it (but see *SI Appendix*, sections 2 and 3 for additional results with ${\tilde{y}}_{\text{mean}}^{*}$ and ${\tilde{y}}_{\text{median}}^{*}$).

With ${\tilde{y}}_{\text{sumlogs}}^{*}$ as the summary statistic, the best-fitting error parameters $\hat{\sigma }$ tended to be smaller for the two-parameter and four-parameter conformity models (all below 0.08) than for the one-stage and two-stage French–DeGroot models (all above 0.2) (*SI Appendix*, Table S3). For all conformity and French–DeGroot models, the best-fitting values of $\hat{\alpha }$ were consistently lower for two of the experimental conditions than the other three, namely $E_{1}$ Dynamic and $E_{2}$ Full Feedback. These are the only two conditions in which participants can both choose whose estimates they view and receive feedback on the accuracy of those estimates. These factors may enable participants to sample more accurate players and have higher confidence in these other players’ opinions relative to their own, lowering $\hat{\alpha }$.

When comparing the best-fitting versions of the conformity and French–DeGroot models to one another, in the case where the summary statistic was ${\tilde{y}}_{\text{sumlogs}}^{*}$, there was a consistent pattern across experimental conditions. For every condition in the full data, subset A data, and subset B data, the conformity models provided a better fit to the data than the French–DeGroot models. A similar result occurred with ${\tilde{y}}_{\text{mean}}^{*}$ in the full data, subset A data, and all but one condition in the subset B data (*SI Appendix*, Table S19), as well as with ${\tilde{y}}_{\text{median}}^{*}$ in subset B. With ${\tilde{y}}_{\text{median}}^{*}$ in the full data and subset A data, the results were more varied (*SI Appendix*, section 3).

In summary, for many, but not all, of our analyses, the conformity models provided a better fit to the data from ref. 37 than the French–DeGroot models. At least one other study has defined conformity with respect to Euclidean distances among choices and investigated it empirically (62). The authors found evidence for conformity under certain conditions of their study using six different distance measures—one of which was the distance to the mean of others’ choices and one of which was the sum of distances to others’ choices. Future research could determine d and k from the data of ref. 62 and compare the fit between different models of conformity and these data.

Many previous studies that have invoked trait-averaging, such as French–DeGroot, models are theoretical rather than empirical. Thus, we investigated whether the conformity model produced fundamentally different theoretical predictions from the French–DeGroot models. In Fig. 2 and *SI Appendix*, Figs. S1, S4, and S5, the conformity model could lead the population to opinions near the extremes of a spectrum at a faster rate than the French–DeGroot models. In Fig. 3 and *SI Appendix*, Fig. S8, the opinion ω=0.9 is considered “correct,” and individuals can adaptively rewire their network connections to increase the chances of sampling an individual with an opinion that is closer to 0.9. In these cases, the conformity model tends to produce a population-average opinion that is farther from the truth (0.9) than the population-average opinion in either of the two French–DeGroot models, reducing the wisdom of the crowd while increasing population-level polarization (*SI Appendix*,Figs. S9 and S10).

Because the conformity model can produce different population-level outcomes from the one- and two-stage French–DeGroot models in these relatively simple examples, it will be interesting to determine whether the conformity model would also change the results of many theoretical studies that have invoked French–DeGroot updating. Future research could assess the extent to which conclusions about information diffusion (48), consensus formation (39, 45, 46), emergency decision-making (49), and a variety of other phenomena from studies based on the French–DeGroot model also hold under the conformity model.

## Supplementary Material

Appendix 01 (PDF)

## Data Availability

Previously published data were used for this work (https://doi.org/10.7910/DVN/EOYZKH) (63). The code used in the present study is available at github.com/kaleda/estimation-biases (64).
