# Supplementary material for: Conformity to popular, not average, opinions: Models, data, and evolution
Source: Proc Natl Acad Sci U S A. 2026 Jun 18;123(25):e2530712123. doi: 10.1073/pnas.2530712123 (PMC13291653; doi:10.1073/pnas.2530712123)
Supplement: Supplementary file 1 — Appendix 01 (PDF) [file pnas.2530712123.sapp.pdf]

PNAS

Supporting Information for

Conformity to popular, not average, opinions: Models, data, and evolution

Kaleda K. Denton[1], Marcus W. Feldman, Jonathan F. Johannemann

[1]**Corresponding Author: Kaleda K. Denton.**
E-mail: kdenton@ucla.edu

This PDF file includes:

Supporting text
Figs. S1 to S10
Tables S1 to S20
SI References

Supporting Information Text

1. Definitions and data sizes

Table S1. Definitions

**Training data**	50% of the data from (1); namely, all odd-numbered rounds of the game with 20 rounds.
**Test data**	50% of the data from (1); namely, all even-numbered rounds of the game with 20 rounds.
**Full data**	All data (either training data or test data, depending on the context) with no subsetting; contrast with subset A and subset B data below.
**Subset A data**	A subset of the data that does not include instances in which individuals did not revise their guess (i.e., their revised guess is identical to their initial guess).
**Subset B data**	A subset of the data in which "low-revision participants" are removed. These are participants whose median extent of revision, namely |revised guess - initial guess|, is less than 0.05.
**Score**	The value of the summary statistic on the data (either training data or test data, depending on the context).
**Trial**	The trial in the optimization procedure (described in SI 2) in which the best-fitting parameters were first obtained.
**Two-parameter conformity model**	The conformity model (see Section 1.B and 1.C in the main text) with σ and α as the only free parameters. d is set to 0.8 and k is set to 2.
**Four-parameter conformity model**	The conformity model (see Section 1.B and 1.C in the main text) with σ, α, d, and k as free parameters.

Table S2. Number of rows of the data for each condition

Data	Condition	Rows in training data	Rows in test data
Full	E_1 Static	2348	2355
	E_1 Dynamic	2330	2327
	E_2 No Feedback	1759	1771
	E_2 Self Feedback	1763	1761
	E_2 Full Feedback	1731	1757
Subset A	E_1 Static	1508	1539
	E_1 Dynamic	1612	1690
	E_2 No Feedback	1075	1031
	E_2 Self Feedback	1043	1094
	E_2 Full Feedback	1247	1312
Subset B	E_1 Static	917	916
	E_1 Dynamic	1168	1169
	E_2 No Feedback	591	597
	E_2 Self Feedback	636	634
	E_2 Full Feedback	967	987

2. Best-fitting parameters

A. Result tables. To obtain the best-fitting parameters for each model, we performed hyperparameter optimization using the `HyperOpt` library (2) in Python. Within this library, we employed a Bayesian optimization algorithm called TPE: Tree-structured Parzen Estimator (3). The output of the function that we sought to optimize (in this case, maximize) was the summary statistic $\widetilde{y}^*$, which could be either $\widetilde{y}^*_{\text{sumlogs}}$, $\widetilde{y}^*_{\text{mean}}$, or $\widetilde{y}^*_{\text{median}}$, described in the main text.

In the optimization algorithm, values of σ were sampled uniformly on [0.0001, 1]. Values of α were sampled log-uniformly on [0.0001, 1000], as log-uniform sampling is better suited than uniform sampling for exploring several orders of magnitude. In principle, α can be even larger than 1000, but we did not view it as qualitatively interesting to determine whether people placed, say, 1000 or 1500 times as much weight on their own guess as on the guesses of others—both numbers are very large. In the four-parameter conformity model (defined in Table S1), values of d were sampled uniformly on [-0.99, 0.99] and values of k were sampled from $\{1, 2, 3\}$.

A set of parameter values was chosen as "best-fitting" if either (i) no further improvement in the fit between the parameters and training data occurred for 300 subsequent trials of the algorithm, or (ii) no further improvement occurred before the maximum trial of 1000 was reached. In the tables below, the "Trial" column gives the trial numbers at which the best-fitting parameter values were first obtained. The fact that these numbers were often considerably less than 1000 indicates that the algorithm was run for sufficiently long to obtain a performance plateau.

In the tables, the best-fitting parameters for each model are denoted with a ˆ symbol. These were obtained using the training data, not the test data, although the score on the test data is also shown in the tables below (see Table S1 for the definition of "score"). High values of $\hat{\alpha}$ (over 20) are highlighted in red; in these cases, individuals placed a large weight $\hat{\alpha}$ on their own opinion relative to 1 for the opinions of others.

Cases in which the score on the test data was lower than it would be under the null model, i.e., the uniform distribution on $[0, 1]$ (namely $\widetilde{y}^*_{\text{sumlogs}} < 0$, $\widetilde{y}^*_{\text{mean}} < 1$, or $\widetilde{y}^*_{\text{median}} < 1$; see Section 2.B of the main text), are highlighted in gray. In these cases, even with their best-fitting parameters, the models do not provide a good fit to the test data. In addition, two-tailed pairwise t-tests and Wilcoxon signed-rank tests comparing the models to the null distribution are given in columns 10 and 11 of the tables, respectively. To answer the question "is the model (in column 1) better than the null?" we write "yes" if the model fits the test data significantly better than the null model (with p-value < 0.05), "no (worse)" if the model provides a significantly worse fit to the test data than the null model (with p-value < 0.05), or "no (same)" if the two models provide similar fits to the data ($p > 0.05$).

For definitions of other terms used in the tables, see Table S1.

Table S3. Results with full data and summary statistic $\widetilde{y}^*_{\text{sumlogs}}$

Model	Condition	$\hat{\sigma}$	$\hat{\alpha}$	$\hat{d}$	$\hat{k}$	Trial	Score on training data	Score on test data	Better than null (t-test)?	Better than null (Wilcoxon)?
One-stage French-DeGroot	E_1 Static	0.26123	5.37	-	-	610	1132.23	1269.69	Yes	Yes
	E_1 Dynamic	0.32547	2.79	-	-	87	1083.07	1038.07	Yes	Yes
	E_2 No Feedback	0.24993	5.66	-	-	401	897.14	985.85	Yes	Yes
	E_2 Self Feedback	0.26098	7.34	-	-	842	786.63	812.20	Yes	Yes
	E_2 Full Feedback	0.34144	3.08	-	-	599	680.04	725.54	Yes	Yes
Two-stage French-DeGroot	E_1 Static	0.27333	10.99	-	-	577	1130.86	1258.53	Yes	Yes
	E_1 Dynamic	0.31772	6.86	-	-	660	1086.37	1047.16	Yes	Yes
	E_2 No Feedback	0.25697	10.94	-	-	232	895.33	986.29	Yes	Yes
	E_2 Self Feedback	0.26157	14.78	-	-	57	785.67	817.70	Yes	Yes
	E_2 Full Feedback	0.35679	6.15	-	-	262	685.84	737.60	Yes	Yes
Two-parameter Conformity	E_1 Static	0.06801	7.49	-	-	212	1998.88	1932.02	Yes	Yes
	E_1 Dynamic	0.07465	3.75	-	-	89	2144.88	1921.00	Yes	Yes
	E_2 No Feedback	0.07672	7.29	-	-	108	1554.67	1686.12	Yes	Yes
	E_2 Self Feedback	0.06231	8.57	-	-	206	1730.18	1549.93	Yes	Yes
	E_2 Full Feedback	0.06695	3.79	-	-	364	1501.88	1357.43	Yes	Yes
Four-parameter Conformity	E_1 Static	0.07612	8.40	0.5952	1	66	2025.51	1941.18	Yes	Yes
	E_1 Dynamic	0.07014	3.74	0.6285	2	788	2168.92	1950.53	Yes	Yes
	E_2 No Feedback	0.07612	8.40	0.5952	1	66	1560.08	1702.69	Yes	Yes
	E_2 Self Feedback	0.06667	6.27	0.6545	1	405	1730.63	1551.56	Yes	Yes
	E_2 Full Feedback	0.06471	3.14	0.5794	2	586	1528.35	1379.15	Yes	Yes

Table S4. Results with full data and summary statistic $\widetilde{y}^*_{\text{mean}}$

Model	Condition	$\hat{\sigma}$	$\hat{\alpha}$	$\hat{d}$	$\hat{k}$	Trial	Score on training data	Score on test data	Better than null (t-test)?	Better than null (Wilcoxon)?
One-stage French-DeGroot	E_1 Static	0.00028	987.23	-	-	744	168.32	187.20	Yes	Yes
	E_1 Dynamic	0.00027	997.43	-	-	165	173.70	149.29	Yes	Yes
	E_2 No Feedback	0.00020	692.55	-	-	102	135.41	167.23	Yes	Yes
	E_2 Self Feedback	0.00010	742.05	-	-	448	170.48	160.24	Yes	Yes
	E_2 Full Feedback	0.00017	936.83	-	-	188	146.30	141.36	Yes	Yes
Two-stage French-DeGroot	E_1 Static	0.00012	971.96	-	-	299	85.62	114.64	Yes	Yes
	E_1 Dynamic	0.00012	672.56	-	-	101	89.37	62.89	Yes	Yes
	E_2 No Feedback	0.00042	982.32	-	-	318	94.13	112.14	Yes	Yes
	E_2 Self Feedback	0.00042	982.32	-	-	318	93.97	92.42	Yes	Yes
	E_2 Full Feedback	0.00035	993.05	-	-	652	76.23	74.03	Yes	Yes
Two-parameter Conformity	E_1 Static	0.00013	29.65	-	-	173	1025.95	987.61	Yes	Yes
	E_1 Dynamic	0.00015	210.01	-	-	942	842.56	732.04	Yes	Yes
	E_2 No Feedback	0.00013	274.27	-	-	760	1178.37	1240.74	Yes	Yes
	E_2 Self Feedback	0.00019	37.84	-	-	296	817.85	746.21	Yes	Yes
	E_2 Full Feedback	0.00031	9.48	-	-	219	294.70	261.56	Yes	Yes
Four-parameter Conformity	E_1 Static	0.00011	22.06	-0.6194	3	91	1132.73	1089.59	Yes	Yes
	E_1 Dynamic	0.00011	22.06	-0.6194	3	91	989.43	861.02	Yes	Yes
	E_2 No Feedback	0.00011	22.06	-0.6194	3	91	1252.00	1321.54	Yes	Yes
	E_2 Self Feedback	0.00011	22.06	-0.6194	3	91	1298.93	1199.43	Yes	Yes
	E_2 Full Feedback	0.00011	22.06	-0.6194	3	91	892.94	801.08	Yes	Yes

Table S5. Results with full data and summary statistic $\widetilde{y}^*_{\text{median}}$

Model	Condition	$\hat{\sigma}$	$\hat{\alpha}$	$\hat{d}$	$\hat{k}$	Trial	Score on training data	Score on test data	Better than null (t-test)?	Better than null (Wilcoxon)?
One-stage French-DeGroot	E_1 Static	0.03719	98.08	-	-	401	7.10	6.53	Yes	Yes
	E_1 Dynamic	0.04613	45.71	-	-	153	5.57	4.84	Yes	Yes
	E_2 No Feedback	0.03333	676.42	-	-	68	7.86	8.22	Yes	Yes
	E_2 Self Feedback	0.02358	144.35	-	-	464	9.69	5.66	Yes	Yes
	E_2 Full Feedback	0.05553	998.98	-	-	465	4.75	3.99	Yes	Yes
Two-stage French-DeGroot	E_1 Static	0.03422	187.92	-	-	634	7.15	6.49	Yes	Yes
	E_1 Dynamic	0.04693	99.15	-	-	394	5.62	4.87	Yes	Yes
	E_2 No Feedback	0.03391	138.39	-	-	178	7.73	9.09	Yes	Yes
	E_2 Self Feedback	0.02596	281.66	-	-	24	9.79	6.27	Yes	Yes
	E_2 Full Feedback	0.05204	981.40	-	-	467	4.73	3.90	Yes	Yes
Two-parameter Conformity	E_1 Static	0.04897	991.11	-	-	65	6.52	5.83	Yes	Yes
	E_1 Dynamic	0.01822	2.12	-	-	795	5.34	3.95	Yes	Yes
	E_2 No Feedback	0.03308	976.26	-	-	296	7.85	7.99	Yes	Yes
	E_2 Self Feedback	0.03817	562.55	-	-	89	8.48	7.43	Yes	Yes
	E_2 Full Feedback	0.05540	993.98	-	-	227	4.70	4.00	Yes	Yes
Four-parameter Conformity	E_1 Static	0.04845	721.00	-0.3297	1	149	6.54	5.84	Yes	Yes
	E_1 Dynamic	0.02202	1.79	0.4579	2	233	4.84	4.03	Yes	Yes
	E_2 No Feedback	0.03223	989.91	-0.6521	1	607	7.99	8.02	Yes	Yes
	E_2 Self Feedback	0.03480	482.46	-0.4587	1	10	8.51	7.84	Yes	Yes
	E_2 Full Feedback	0.01541	1.77	0.2691	2	744	5.01	4.12	Yes	Yes

Table S6. Results with subset A data and summary statistic $\widetilde{y}^{*}_{\text{sumlogs}}$

Model	Condition	$\hat{\sigma}$	$\hat{\alpha}$	$\hat{d}$	$\hat{k}$	Trial	Score on training data	Score on test data	Better than null (t-test)?	Better than null (Wilcoxon)?
One-stage French-DeGroot	E_1 Static	0.35764	1.95	-	-	424	584.42	669.20	Yes	Yes
	E_1 Dynamic	0.36046	1.14	-	-	843	714.70	726.08	Yes	Yes
	E_2 No Feedback	0.34532	1.80	-	-	358	462.03	476.18	Yes	Yes
	E_2 Self Feedback	0.37498	2.13	-	-	159	356.40	410.26	Yes	Yes
	E_2 Full Feedback	0.39021	1.16	-	-	437	456.96	501.36	Yes	Yes
Two-stage French-DeGroot	E_1 Static	0.37604	4.36	-	-	96	591.82	681.01	Yes	Yes
	E_1 Dynamic	0.38743	2.86	-	-	247	716.02	746.54	Yes	Yes
	E_2 No Feedback	0.37301	4.16	-	-	298	465.28	474.01	Yes	Yes
	E_2 Self Feedback	0.40431	4.48	-	-	442	360.41	418.28	Yes	Yes
	E_2 Full Feedback	0.44622	2.29	-	-	643	475.51	514.21	Yes	Yes
Two-parameter Conformity	E_1 Static	0.10283	2.81	-	-	31	874.45	829.11	Yes	Yes
	E_1 Dynamic	0.08423	1.52	-	-	342	1168.55	1118.94	Yes	Yes
	E_2 No Feedback	0.10573	4.05	-	-	321	643.09	654.78	Yes	Yes
	E_2 Self Feedback	0.09631	2.58	-	-	32	686.38	626.05	Yes	Yes
	E_2 Full Feedback	0.08580	1.19	-	-	641	870.88	802.37	Yes	Yes
Four-parameter Conformity	E_1 Static	0.10913	2.88	0.6015	3	79	903.17	869.04	Yes	Yes
	E_1 Dynamic	0.08685	1.33	0.7386	3	641	1197.96	1133.35	Yes	Yes
	E_2 No Feedback	0.10411	3.43	0.7390	3	320	657.61	672.10	Yes	Yes
	E_2 Self Feedback	0.09438	2.47	0.6640	3	82	694.50	646.88	Yes	Yes
	E_2 Full Feedback	0.09444	1.27	0.3846	2	96	877.97	800.91	Yes	Yes

Table S7. Results with subset A data and summary statistic $\widetilde{y}^{*}_{\text{mean}}$

Model	Condition	$\hat{\sigma}$	$\hat{\alpha}$	$\hat{d}$	$\hat{k}$	Trial	Score on training data	Score on test data	Better than null (t-test)?	Better than null (Wilcoxon)?
One-stage French-DeGroot	E_1 Static	0.00010	21.96	-	-	401	9.98	7.22	No (same)	No (worse)
	E_1 Dynamic	0.00024	0.00	-	-	617	33.59	27.84	Yes	No (worse)
	E_2 No Feedback	0.00011	5.80	-	-	109	4.72	0.27	No (worse)	No (worse)
	E_2 Self Feedback	0.00021	42.31	-	-	305	5.50	0.90	No (same)	No (worse)
	E_2 Full Feedback	0.00010	0.00	-	-	91	49.88	60.22	Yes	No (worse)
Two-stage French-DeGroot	E_1 Static	0.00123	35.65	-	-	160	4.23	4.24	Yes	No (worse)
	E_1 Dynamic	0.00011	23.01	-	-	401	9.25	6.36	No (same)	No (worse)
	E_2 No Feedback	0.00016	0.23	-	-	288	14.63	0.00	No (worse)	No (worse)
	E_2 Self Feedback	0.00011	31.48	-	-	777	11.86	8.67	No (same)	No (worse)
	E_2 Full Feedback	0.00015	0.00	-	-	188	9.10	0.00	No (worse)	No (worse)
Two-parameter Conformity	E_1 Static	0.00011	0.00	-	-	91	123.22	130.78	Yes	Yes
	E_1 Dynamic	0.00011	0.00	-	-	91	192.13	136.80	Yes	Yes
	E_2 No Feedback	0.00011	0.00	-	-	91	150.65	174.45	Yes	Yes
	E_2 Self Feedback	0.00011	0.00	-	-	91	158.60	104.90	Yes	No (same)
	E_2 Full Feedback	0.00011	0.00	-	-	91	194.22	169.80	Yes	Yes
Four-parameter Conformity	E_1 Static	0.00014	0.05	0.9894	2	349	101.78	106.72	Yes	Yes
	E_1 Dynamic	0.00011	0.01	0.4133	1	91	174.06	123.67	Yes	Yes
	E_2 No Feedback	0.00016	0.79	0.4201	1	188	78.42	86.92	Yes	Yes
	E_2 Self Feedback	0.00010	0.00	0.2926	2	817	148.58	102.18	Yes	No (same)
	E_2 Full Feedback	0.00011	0.01	0.4133	1	91	175.39	158.59	Yes	Yes

Table S8. Results with subset A data and summary statistic $\widetilde{y}^*_{\text{median}}$

Model	Condition	$\hat{\sigma}$	$\hat{\alpha}$	$\hat{d}$	$\hat{k}$	Trial	Score on training data	Score on test data	Better than null (t-test)?	Better than null (Wilcoxon)?
One-stage French-DeGroot	E_1 Static	0.11096	13.66	-	-	276	2.95	3.10	Yes	Yes
	E_1 Dynamic	0.12598	10.47	-	-	260	2.56	2.73	Yes	Yes
	E_2 No Feedback	0.10526	8.77	-	-	146	3.06	3.29	Yes	Yes
	E_2 Self Feedback	0.10086	18.92	-	-	667	2.87	2.93	Yes	Yes
	E_2 Full Feedback	0.13648	10.68	-	-	273	2.44	2.49	Yes	Yes
Two-stage French-DeGroot	E_1 Static	0.11083	27.42	-	-	333	2.96	3.09	Yes	Yes
	E_1 Dynamic	0.13763	15.99	-	-	65	2.58	2.68	Yes	Yes
	E_2 No Feedback	0.11623	16.54	-	-	91	3.02	3.22	Yes	Yes
	E_2 Self Feedback	0.09961	41.19	-	-	271	2.83	2.95	Yes	Yes
	E_2 Full Feedback	0.12962	24.16	-	-	224	2.45	2.50	Yes	Yes
Two-parameter Conformity	E_1 Static	0.08947	973.43	-	-	178	2.68	2.56	Yes	Yes
	E_1 Dynamic	0.04708	0.00	-	-	547	3.14	2.78	Yes	Yes
	E_2 No Feedback	0.07658	2.98	-	-	226	2.55	2.63	Yes	Yes
	E_2 Self Feedback	0.03813	0.94	-	-	39	2.90	2.27	Yes	Yes
	E_2 Full Feedback	0.04854	0.01	-	-	221	3.06	2.63	Yes	Yes
Four-parameter Conformity	E_1 Static	0.08802	773.24	0.2306	2	238	2.68	2.51	Yes	Yes
	E_1 Dynamic	0.04642	0.00	0.9223	2	71	3.19	2.86	Yes	Yes
	E_2 No Feedback	0.08634	22.22	0.9887	3	821	2.66	2.84	Yes	Yes
	E_2 Self Feedback	0.03763	0.96	0.8057	2	407	2.91	2.28	Yes	Yes
	E_2 Full Feedback	0.04561	0.75	0.9466	2	536	3.13	2.70	Yes	Yes

Table S9. Results with subset B data and summary statistic $\widetilde{y}^*_{\text{sumlogs}}$

Model	Condition	$\hat{\sigma}$	$\hat{\alpha}$	$\hat{d}$	$\hat{k}$	Trial	Score on training data	Score on test data	Better than null (t-test)?	Better than null (Wilcoxon)?
One-stage French-DeGroot	E_1 Static	0.38962	1.11	-	-	262	329.85	377.52	Yes	Yes
	E_1 Dynamic	0.36855	0.92	-	-	32	477.81	418.13	Yes	Yes
	E_2 No Feedback	0.33355	1.75	-	-	430	238.82	255.98	Yes	Yes
	E_2 Self Feedback	0.42857	1.30	-	-	555	186.23	202.15	Yes	Yes
	E_2 Full Feedback	0.41104	0.93	-	-	864	330.60	366.29	Yes	Yes
Two-stage French-DeGroot	E_1 Static	0.45669	3.21	-	-	671	326.23	379.03	Yes	Yes
	E_1 Dynamic	0.44442	2.80	-	-	307	472.16	434.92	Yes	Yes
	E_2 No Feedback	0.37604	4.36	-	-	96	237.88	248.28	Yes	Yes
	E_2 Self Feedback	0.45867	3.72	-	-	243	183.06	208.30	Yes	Yes
	E_2 Full Feedback	0.46764	2.82	-	-	898	329.72	376.89	Yes	Yes
Two-parameter Conformity	E_1 Static	0.09705	1.95	-	-	428	529.18	556.43	Yes	Yes
	E_1 Dynamic	0.08054	1.57	-	-	401	865.56	776.48	Yes	Yes
	E_2 No Feedback	0.09631	2.88	-	-	32	385.75	406.26	Yes	Yes
	E_2 Self Feedback	0.08686	1.72	-	-	462	428.53	378.87	Yes	Yes
	E_2 Full Feedback	0.08206	1.27	-	-	585	697.08	645.73	Yes	Yes
Four-parameter Conformity	E_1 Static	0.10728	1.32	0.4228	2	258	543.27	529.75	Yes	Yes
	E_1 Dynamic	0.07500	1.68	0.8034	3	474	885.31	776.56	Yes	Yes
	E_2 No Feedback	0.09438	2.47	0.6640	3	82	388.39	415.01	Yes	Yes
	E_2 Self Feedback	0.08097	1.40	0.6192	2	789	430.06	390.05	Yes	Yes
	E_2 Full Feedback	0.07225	1.25	0.3634	2	97	704.43	654.51	Yes	Yes

Table S10. Results with subset B data and summary statistic $\widetilde{y}^*_{\text{mean}}$

Model	Condition	$\hat{\sigma}$	$\hat{\alpha}$	$\hat{d}$	$\hat{k}$	Trial	Score on training data	Score on test data	Better than null (t-test)?	Better than null (Wilcoxon)?
One-stage French-DeGroot	E_1 Static	0.00030	987.34	-	-	398	100.35	115.06	Yes	Yes
	E_1 Dynamic	0.00021	0.00	-	-	149	50.19	32.52	Yes	No (worse)
	E_2 No Feedback	0.00054	999.93	-	-	119	79.50	108.29	Yes	Yes
	E_2 Self Feedback	0.00018	996.22	-	-	955	115.50	106.14	Yes	Yes
	E_2 Full Feedback	0.00010	0.00	-	-	91	71.43	53.30	Yes	No (worse)
Two-stage French-DeGroot	E_1 Static	0.00014	996.46	-	-	628	66.93	95.44	Yes	Yes
	E_1 Dynamic	0.00021	981.43	-	-	157	65.19	48.40	Yes	Yes
	E_2 No Feedback	0.00017	919.34	-	-	288	44.64	74.91	Yes	Yes
	E_2 Self Feedback	0.00020	987.81	-	-	620	57.99	56.30	Yes	Yes
	E_2 Full Feedback	0.00012	989.11	-	-	501	58.94	50.71	Yes	No (same)
Two-parameter Conformity	E_1 Static	0.00011	112.39	-	-	760	649.75	629.06	Yes	Yes
	E_1 Dynamic	0.00011	0.00	-	-	91	185.03	144.86	Yes	Yes
	E_2 No Feedback	0.00025	251.14	-	-	297	294.57	356.89	Yes	Yes
	E_2 Self Feedback	0.00011	0.00	-	-	91	169.60	128.76	Yes	Yes
	E_2 Full Feedback	0.00011	0.00	-	-	91	215.82	187.17	Yes	Yes
Four-parameter Conformity	E_1 Static	0.00011	22.06	-0.6194	3	91	592.02	574.35	Yes	Yes
	E_1 Dynamic	0.00013	19.04	-0.3212	2	359	495.21	436.95	Yes	Yes
	E_2 No Feedback	0.00011	22.06	-0.6194	3	91	575.88	692.39	Yes	Yes
	E_2 Self Feedback	0.00013	130.68	-0.4846	2	359	621.45	606.78	Yes	Yes
	E_2 Full Feedback	0.00013	1.18	-0.1879	1	359	246.92	218.96	Yes	Yes

Table S11. Results with subset B data and summary statistic $\widetilde{y}^*_{\text{median}}$

Model	Condition	$\hat{\sigma}$	$\hat{\alpha}$	$\hat{d}$	$\hat{k}$	Trial	Score on training data	Score on test data	Better than null (t-test)?	Better than null (Wilcoxon)?
One-stage French-DeGroot	E_1 Static	0.13078	10.96	-	-	401	2.47	2.66	Yes	Yes
	E_1 Dynamic	0.12704	11.97	-	-	461	2.43	2.41	Yes	Yes
	E_2 No Feedback	0.10054	17.89	-	-	562	2.85	2.74	Yes	Yes
	E_2 Self Feedback	0.13798	12.41	-	-	452	2.33	2.56	Yes	Yes
	E_2 Full Feedback	0.19546	0.00	-	-	291	2.14	1.95	Yes	Yes
Two-stage French-DeGroot	E_1 Static	0.13926	23.01	-	-	429	2.44	2.62	Yes	Yes
	E_1 Dynamic	0.12027	28.70	-	-	297	2.40	2.45	Yes	Yes
	E_2 No Feedback	0.11139	29.15	-	-	533	2.86	2.80	Yes	Yes
	E_2 Self Feedback	0.13847	27.21	-	-	409	2.32	2.55	Yes	Yes
	E_2 Full Feedback	0.12777	29.54	-	-	133	2.27	2.32	Yes	Yes
Two-parameter Conformity	E_1 Static	0.03813	0.94	-	-	39	2.84	2.92	Yes	Yes
	E_1 Dynamic	0.02574	0.79	-	-	280	3.70	3.00	Yes	Yes
	E_2 No Feedback	0.01835	1.19	-	-	786	3.39	3.47	Yes	Yes
	E_2 Self Feedback	0.03946	0.00	-	-	37	2.98	2.23	Yes	Yes
	E_2 Full Feedback	0.01972	1.32	-	-	439	3.84	2.75	Yes	Yes
Four-parameter Conformity	E_1 Static	0.07303	1.54	0.9898	1	436	2.84	2.57	Yes	Yes
	E_1 Dynamic	0.02094	0.86	0.1188	1	553	3.74	3.25	Yes	Yes
	E_2 No Feedback	0.01434	0.78	0.3924	1	172	2.98	3.17	Yes	Yes
	E_2 Self Feedback	0.02772	1.59	0.9540	2	585	3.58	2.84	Yes	Yes
	E_2 Full Feedback	0.02244	1.08	0.9018	3	408	3.90	2.81	Yes	Yes

B. Summary and discussion of the above results. For a given model (e.g., conformity or French-DeGroot), the best-fitting parameter values often vary considerably across different summary statistics that are maximized by the optimization algorithm. There is less variation in best-fitting parameter values across different experimental conditions.

Consider the one- and two-stage French-DeGroot models. When the summary statistic is $\widetilde{y}^*_{\text{mean}}$, for all experimental conditions, the best-fitting parameters are $\hat{\alpha} > 670$ and $\hat{\sigma} < 0.0005$ (Table S4). With $\widetilde{y}^*_{\text{median}}$, $\hat{\alpha} > 45$ and $\hat{\sigma} < 0.06$ for all experimental conditions (Table S5). However, with $\widetilde{y}^*_{\text{sumlogs}}$, $\hat{\alpha}$ is less than 15, often closer to 5 or 6 (Table S3). The conformity models show a similar, but less pronounced, difference in $\hat{\alpha}$ values between $\widetilde{y}^*_{\text{mean}}$ or $\widetilde{y}^*_{\text{median}}$ and $\widetilde{y}^*_{\text{sumlogs}}$ (Table S3-Table S5).

We conducted two follow-up analyses to explore why some of the best-fitting $\hat{\alpha}$ values were so high, particularly for $\widetilde{y}^*_{\text{mean}}$. High $\hat{\alpha}$ values suggest that individuals place a much larger weight on their own opinions than those of others. However, it is possible that a small number of data points with extremely large y^* values—for example, cases where individuals did not revise their guess at all and $\hat{\alpha}$ was large—could 'dominate,' i.e., greatly affect, the calculation of $\widetilde{y}^*_{\text{mean}}$. To explore the consequences of removing certain data points, we partitioned the data in two ways, referred to as subsets A and B.

In subset A, we omitted instances in which individuals' revised guesses were identical to their initial guesses. If removing such cases caused $\hat{\alpha}$ values to go from high to low, it would seem that when individuals did revise their guesses, they tended to weigh the opinions of others considerably. Indeed, compared to the full data, in subset A data with $\widetilde{y}^*_{\text{mean}}$ as the summary statistic, $\hat{\alpha}$ values tended to be much lower—even approximately zero for some conditions (Table S7). Similarly, with $\widetilde{y}^*_{\text{sumlogs}}$ and $\widetilde{y}^*_{\text{median}}$, $\hat{\alpha}$ values were almost always lower for subset A data than for the full data (Table S6 and Table S8).

In subset B, we excluded players who tended not to revise their guesses very much across the 20 rounds of the game (although in any given round, they could revise their guess by a non-zero amount). Specifically, we removed "low-revision players" whose typical (median) extent of revision across the 20 rounds of the game was less than 0.05 (Table S1). Recall that the scatter plot correlation that individuals were estimating could range from 0 to 1, so a difference in |revised guess - initial guess| of 0.05 or less is fairly small. In subset B, best-fitting $\hat{\alpha}$ values remained high for some conditions (e.g., often when the summary statistic was $\widetilde{y}^*_{\text{mean}}$; Table S10) but decreased relative to the full data under other conditions (e.g., for $\widetilde{y}^*_{\text{sumlogs}}$ and $\widetilde{y}^*_{\text{median}}$; Table S9 and Table S11).

Overall, when the summary statistic was $\widetilde{y}^*_{\text{mean}}$, high $\hat{\alpha}$ values seem to be primarily driven by cases in which individuals' revised guesses were identical to their initial guesses—as their removal in subset A caused $\hat{\alpha}$ values to decrease significantly—rather than "low-revision players," whose removal was explored in subset B. When the summary statistic was $\widetilde{y}^*_{\text{median}}$, both cases of zero-revision and low-revision players contributed to high $\hat{\alpha}$ values. The same was true for $\widetilde{y}^*_{\text{sumlogs}}$, although in these cases, $\hat{\alpha}$ values were not as high to begin with (i.e., in the full data; Table S3) compared to $\widetilde{y}^*_{\text{mean}}$ or $\widetilde{y}^*_{\text{median}}$.

In examining the 15 conditions (3 summary statistics $\times$ 5 experimental conditions) in the 3 data types (full data, subset A data, and subset B data), for a total of 45 analyses, an interesting pattern appeared in the conformity model with d as a free parameter. Of the 13 cases in which $\hat{\alpha} > 20$, highlighted in red in SI 2.A, $\hat{d}$ was negative in 11 cases and positive in 2 cases. Conversely, of the 32 cases in which $\hat{\alpha} < 20$ (31 of which $\hat{\alpha} < 9$), $\hat{d}$ was positive in 30 cases and negative in 2 cases—one of which had $\hat{\alpha} \approx 19$. The best-fitting $\hat{d}$ parameter was not found to be zero, which would have indicated random copying, in any of the cases.

The finding that high $\hat{\alpha}$ values were strongly associated with negative $\hat{d}$ values makes intuitive sense. Recall that in cases where $\hat{\alpha}$ is high, individuals tend to adhere to their original opinions rather than adopting the opinions of others, so $\hat{d}$, which governs conformity or anti-conformity to others' opinions, does not play a large role in the outcome of the model. Nevertheless, one would expect that in cases where people disregard the opinions of others, anti-conformity ($\hat{d} < 0$), rather than conformity ($\hat{d} > 0$) to others' opinions would be the best-fitting $\hat{d}$ parameter, which is what we found. In the cases with smaller $\hat{\alpha}$, where $\hat{d}$ plays a larger role in the model, conformity ($\hat{d} > 0$) was almost always found to be a better fit to the data than anti-conformity ($\hat{d} < 0$).

Finally, all of the French-DeGroot and conformity models, with their best-fitting parameters, provided a better fit to the full test data than the null distribution (Table S3-Table S5). The same was true for subset A and subset B data when the summary statistic was $\widetilde{y}^*_{\text{sumlogs}}$ or $\widetilde{y}^*_{\text{median}}$ (Table S6, Table S8, Table S9, Table S11). For subset A data with summary statistic $\widetilde{y}^*_{\text{mean}}$, the null distribution often fit the data better than the French-DeGroot models, whereas the conformity models provided a better or, in some rare cases, similar fit to the data than the null distribution (Table S7). Finally, in subset B data with summary statistic $\widetilde{y}^*_{\text{mean}}$, the null distribution fit the data better than the one-stage French-DeGroot model under some conditions, whereas the two-stage French-DeGroot model often, and conformity models always, fit the data better than the null distribution (Table S10).

3. Comparing the best-fitting French-DeGroot and conformity models

A. Result tables. In the tables below, "FDG" stands for French-DeGroot, "Conformity-2" stands for the conformity model with two free parameters, and "Conformity-4" stands for the conformity model with 4 free parameters. For each data type (full, subset A, and subset B, defined in Table S1), each summary statistic ($\widetilde{y}^*_{\text{sumlogs}}$, $\widetilde{y}^*_{\text{mean}}$, and $\widetilde{y}^*_{\text{median}}$), and each experimental condition (E_1 Static, E_1 Dynamic, E_2 No Feedback, E_2 Self Feedback, and E_2 Full Feedback), statistical tests were used to compare the "first model" (column 1 of the tables) to the "second model" (column 2), both with their parameters taking the best-fitting values from SI 2. The best-fitting $\hat{\alpha}$ values are reproduced in the tables below for ease of comparison.

The statistical tests included two-tailed, pairwise t-tests and two-tailed Wilcoxon signed-rank tests. These tests were performed on the y^* values of each model—see Figure 1 from the main text for examples—averaged over each participant, whose multiple y^* values constituted dependent observations. In the t-test, a positive t-statistic implies that the first model (column 1) produces higher y^* values than the second model (column 2), meaning that the first model is a better fit to the data, whereas a negative t-statistic implies that the second model is a better fit to the data. The t-statistics are given in the sixth column of the tables below. The difference between models is said to be significant if the p value is less than 0.05 (seventh column).

In the Wilcoxon test, we assessed which model—the first or the second—produced a better fit to the data as follows. First, we computed pairwise differences in participant-averaged y^* values between the models, denoted by δ. Second, after discarding values that were zero, we ranked the absolute values of the differences, $|\delta|$, where rank 1 corresponds to the smallest $|\delta|$, rank 2 corresponds to the second-smallest $|\delta|$, and so on. Third, we obtained signed ranks by assigning each rank $(1, 2, \dots)$ the sign ($+$ or $-$) of the original δ to which it corresponded. Finally, we summed all of these signed ranks to determine the signed-rank sum, W, which is the Wilcoxon test statistic. If $W > 0$, then the first model (column 1) tended to have higher y^* values than the second model (column 2), whereas if $W < 0$, the opposite was true. In cases where one model provided a significantly better fit to the data according to *both* the t-test and the Wilcoxon test, it is highlighted in green.

Table S12. Model comparisons with full data and summary statistic $\widetilde{y}^{*}_{\text{sumlogs}}$. The best-fitting parameters for these models are given in Table S3, and $\hat{\alpha}$ values are repeated below.

First model	Second model	Condition	First model $\hat{\alpha}$	Second model $\hat{\alpha}$	t-statistic	t-test p value	Wilcoxon statistic	Wilcoxon p value
One-stage FDG	Two-stage FDG	E_1 Static	5.37	10.99	21.2793	8.2×10^{-57}	26677	1.6×10^{-36}
One-stage FDG	Two-stage FDG	E_1 Dynamic	2.79	6.86	-13.8559	2.6×10^{-32}	-22290	2.5×10^{-26}
One-stage FDG	Two-stage FDG	E_2 No Feedback	5.66	10.94	2.8504	4.9×10^{-3}	2304	1.0×10^{-1}
One-stage FDG	Two-stage FDG	E_2 Self Feedback	7.34	14.78	-13.4039	9.9×10^{-29}	-13587	5.7×10^{-23}
One-stage FDG	Two-stage FDG	E_2 Full Feedback	3.08	6.15	-2.8499	4.9×10^{-3}	-3475	1.2×10^{-2}
One-stage FDG	Conformity-2	E_1 Static	5.37	7.49	-34.1694	2.4×10^{-93}	-28193	1.3×10^{-40}
One-stage FDG	Conformity-2	E_1 Dynamic	2.79	3.75	-38.0639	1.8×10^{-102}	-27934	2.2×10^{-40}
One-stage FDG	Conformity-2	E_2 No Feedback	5.66	7.29	-32.9242	7.3×10^{-78}	-16290	2.7×10^{-31}
One-stage FDG	Conformity-2	E_2 Self Feedback	7.34	8.57	-31.5489	1.4×10^{-74}	-15915	6.7×10^{-31}
One-stage FDG	Conformity-2	E_2 Full Feedback	3.08	3.79	-31.5130	1.7×10^{-74}	-15921	6.3×10^{-31}
One-stage FDG	Conformity-4	E_1 Static	5.37	8.40	-32.5073	4.0×10^{-89}	-28163	1.6×10^{-40}
One-stage FDG	Conformity-4	E_1 Dynamic	2.79	3.74	-36.9311	8.0×10^{-100}	-27908	2.6×10^{-40}
One-stage FDG	Conformity-4	E_2 No Feedback	5.66	8.40	-31.1829	2.9×10^{-74}	-16286	2.8×10^{-31}
One-stage FDG	Conformity-4	E_2 Self Feedback	7.34	6.27	-31.2531	5.8×10^{-74}	-15909	7.0×10^{-31}
One-stage FDG	Conformity-4	E_2 Full Feedback	3.08	3.14	-31.4840	1.9×10^{-74}	-15927	6.0×10^{-31}
Two-stage FDG	Conformity-2	E_1 Static	10.99	7.49	-34.2423	1.6×10^{-93}	-28193	1.3×10^{-40}
Two-stage FDG	Conformity-2	E_1 Dynamic	6.86	3.75	-37.4048	6.2×10^{-101}	-27916	2.5×10^{-40}
Two-stage FDG	Conformity-2	E_2 No Feedback	10.94	7.29	-32.3085	1.3×10^{-76}	-16290	2.7×10^{-31}
Two-stage FDG	Conformity-2	E_2 Self Feedback	14.78	8.57	-31.2989	4.6×10^{-74}	-15913	6.8×10^{-31}
Two-stage FDG	Conformity-2	E_2 Full Feedback	6.15	3.79	-30.6478	1.1×10^{-72}	-15917	6.6×10^{-31}
Two-stage FDG	Conformity-4	E_1 Static	10.99	8.40	-32.5363	3.4×10^{-89}	-28169	1.5×10^{-40}
Two-stage FDG	Conformity-4	E_1 Dynamic	6.86	3.74	-36.5112	7.9×10^{-99}	-27894	2.9×10^{-40}
Two-stage FDG	Conformity-4	E_2 No Feedback	10.94	8.40	-30.4036	1.3×10^{-72}	-16284	2.9×10^{-31}
Two-stage FDG	Conformity-4	E_2 Self Feedback	14.78	6.27	-30.9380	2.6×10^{-73}	-15909	7.0×10^{-31}
Two-stage FDG	Conformity-4	E_2 Full Feedback	6.15	3.14	-30.5408	1.8×10^{-72}	-15925	6.1×10^{-31}

Table S13. Model comparisons with full data and summary statistic $\widetilde{y}^*_{\text{mean}}$. The best-fitting parameters for these models are given in Table S4, and $\hat{\alpha}$ values are repeated below.

First model	Second model	Condition	First model $\hat{\alpha}$	Second model $\hat{\alpha}$	t-statistic	t-test p value	Wilcoxon statistic	Wilcoxon p value
One-stage FDG	Two-stage FDG	E_1 Static	987.23	971.96	8.3122	7.5×10^{-15}	14160	1.4×10^{-14}
One-stage FDG	Two-stage FDG	E_1 Dynamic	997.43	672.56	10.7704	3.0×10^{-22}	17678	4.9×10^{-23}
One-stage FDG	Two-stage FDG	E_2 No Feedback	692.55	982.32	8.1823	5.0×10^{-14}	8479	1.3×10^{-10}
One-stage FDG	Two-stage FDG	E_2 Self Feedback	742.05	982.32	4.2491	3.5×10^{-5}	1820	1.6×10^{-1}
One-stage FDG	Two-stage FDG	E_2 Full Feedback	936.83	993.05	10.7324	5.1×10^{-21}	10665	2.1×10^{-20}
One-stage FDG	Conformity-2	E_1 Static	987.23	29.65	-21.4249	2.8×10^{-57}	-27026	8.3×10^{-40}
One-stage FDG	Conformity-2	E_1 Dynamic	997.43	210.01	-19.2604	3.0×10^{-50}	-24956	2.8×10^{-38}
One-stage FDG	Conformity-2	E_2 No Feedback	692.55	274.27	-20.6085	3.9×10^{-49}	-15576	1.2×10^{-30}
One-stage FDG	Conformity-2	E_2 Self Feedback	742.05	37.84	-15.6112	4.1×10^{-35}	-13887	3.3×10^{-27}
One-stage FDG	Conformity-2	E_2 Full Feedback	936.83	9.48	-9.5986	7.9×10^{-18}	-11873	3.4×10^{-18}
One-stage FDG	Conformity-4	E_1 Static	987.23	22.06	-21.0199	5.5×10^{-56}	-27026	8.3×10^{-40}
One-stage FDG	Conformity-4	E_1 Dynamic	997.43	22.06	-19.4427	7.8×10^{-51}	-24956	2.8×10^{-38}
One-stage FDG	Conformity-4	E_2 No Feedback	692.55	22.06	-20.4644	9.6×10^{-49}	-15576	1.2×10^{-30}
One-stage FDG	Conformity-4	E_2 Self Feedback	742.05	22.06	-16.8339	1.4×10^{-38}	-14487	1.8×10^{-29}
One-stage FDG	Conformity-4	E_2 Full Feedback	936.83	22.06	-15.3035	3.2×10^{-34}	-14706	8.2×10^{-30}
Two-stage FDG	Conformity-2	E_1 Static	971.96	29.65	-20.8038	2.7×10^{-55}	-26744	1.7×10^{-39}
Two-stage FDG	Conformity-2	E_1 Dynamic	672.56	210.01	-18.9750	2.6×10^{-49}	-23916	3.6×10^{-37}
Two-stage FDG	Conformity-2	E_2 No Feedback	982.32	274.27	-21.1041	1.9×10^{-50}	-16098	4.4×10^{-31}
Two-stage FDG	Conformity-2	E_2 Self Feedback	982.32	37.84	-18.7235	7.7×10^{-44}	-15205	3.1×10^{-30}
Two-stage FDG	Conformity-2	E_2 Full Feedback	993.05	9.48	-15.4450	1.2×10^{-34}	-15683	1.5×10^{-30}
Two-stage FDG	Conformity-4	E_1 Static	971.96	22.06	-20.5716	1.5×10^{-54}	-26702	2.2×10^{-39}
Two-stage FDG	Conformity-4	E_1 Dynamic	672.56	22.06	-19.2378	3.6×10^{-50}	-24074	1.2×10^{-37}
Two-stage FDG	Conformity-4	E_2 No Feedback	982.32	22.06	-20.9185	5.9×10^{-50}	-16098	4.4×10^{-31}
Two-stage FDG	Conformity-4	E_2 Self Feedback	982.32	22.06	-18.0853	4.4×10^{-42}	-15205	3.1×10^{-30}
Two-stage FDG	Conformity-4	E_2 Full Feedback	993.05	22.06	-16.0220	2.8×10^{-36}	-15045	4.1×10^{-30}

 Kaleda K. Denton[1], Marcus W. Feldman, Jonathan F. Johannemann

Table S14. Model comparisons with full data and summary statistic $\widetilde{y}^*_{\text{median}}$. The best-fitting parameters for these models are given in Table S5, and $\hat{\alpha}$ values are repeated below.

First model	Second model	Condition	First model $\hat{\alpha}$	Second model $\hat{\alpha}$	t-statistic	t-test p value	Wilcoxon statistic	Wilcoxon p value
One-stage FDG	Two-stage FDG	E_1 Static	98.08	187.92	-21.2818	8.0×10^{-57}	-27301	3.5×10^{-38}
One-stage FDG	Two-stage FDG	E_1 Dynamic	45.71	99.15	16.1466	5.8×10^{-40}	24534	1.5×10^{-31}
One-stage FDG	Two-stage FDG	E_2 No Feedback	676.42	138.39	1.9664	5.1×10^{-2}	2080	1.4×10^{-1}
One-stage FDG	Two-stage FDG	E_2 Self Feedback	144.35	281.66	16.1851	9.5×10^{-37}	15381	5.7×10^{-29}
One-stage FDG	Two-stage FDG	E_2 Full Feedback	998.98	981.40	-17.9466	1.1×10^{-41}	-15461	2.9×10^{-29}
One-stage FDG	Conformity-2	E_1 Static	98.08	991.11	23.0007	3.3×10^{-62}	27655	3.9×10^{-39}
One-stage FDG	Conformity-2	E_1 Dynamic	45.71	2.12	-10.0928	3.9×10^{-20}	-18034	8.8×10^{-18}
One-stage FDG	Conformity-2	E_2 No Feedback	676.42	976.26	1.1876	2.4×10^{-1}	1010	4.7×10^{-1}
One-stage FDG	Conformity-2	E_2 Self Feedback	144.35	562.55	17.6605	6.7×10^{-41}	15601	9.2×10^{-30}
One-stage FDG	Conformity-2	E_2 Full Feedback	998.98	993.98	2.8690	4.6×10^{-3}	3723	6.9×10^{-3}
One-stage FDG	Conformity-4	E_1 Static	98.08	721.00	23.1095	1.5×10^{-62}	27677	3.4×10^{-39}
One-stage FDG	Conformity-4	E_1 Dynamic	45.71	1.79	-2.2421	2.6×10^{-2}	-3294	1.2×10^{-1}
One-stage FDG	Conformity-4	E_2 No Feedback	676.42	989.91	-14.6046	2.6×10^{-32}	-14656	1.2×10^{-25}
One-stage FDG	Conformity-4	E_2 Self Feedback	144.35	482.46	17.8515	2.0×10^{-41}	15603	9.1×10^{-30}
One-stage FDG	Conformity-4	E_2 Full Feedback	998.98	1.77	-13.1971	3.9×10^{-28}	-13743	1.8×10^{-23}
Two-stage FDG	Conformity-2	E_1 Static	187.92	991.11	22.6828	3.2×10^{-61}	27599	5.5×10^{-39}
Two-stage FDG	Conformity-2	E_1 Dynamic	99.15	2.12	-10.5333	1.7×10^{-21}	-18624	7.4×10^{-19}
Two-stage FDG	Conformity-2	E_2 No Feedback	138.39	976.26	-1.7195	8.7×10^{-2}	-2028	1.5×10^{-1}
Two-stage FDG	Conformity-2	E_2 Self Feedback	281.66	562.55	18.0295	6.3×10^{-42}	15623	7.7×10^{-30}
Two-stage FDG	Conformity-2	E_2 Full Feedback	981.40	993.98	16.9592	6.1×10^{-39}	15193	2.6×10^{-28}
Two-stage FDG	Conformity-4	E_1 Static	187.92	721.00	22.7619	1.8×10^{-61}	27633	4.5×10^{-39}
Two-stage FDG	Conformity-4	E_1 Dynamic	99.15	1.79	-2.7617	6.2×10^{-3}	-4370	3.7×10^{-2}
Two-stage FDG	Conformity-4	E_2 No Feedback	138.39	989.91	-4.6621	6.1×10^{-6}	-6310	6.6×10^{-6}
Two-stage FDG	Conformity-4	E_2 Self Feedback	281.66	482.46	18.3556	7.9×10^{-43}	15631	7.2×10^{-30}
Two-stage FDG	Conformity-4	E_2 Full Feedback	981.40	1.77	-11.9391	1.8×10^{-24}	-12991	3.9×10^{-21}

Table S15. Model comparisons with subset A data and summary statistic $\widetilde{y}^{*}_{\text{sumlogs}}$. The best-fitting parameters for these models are given in Table S6, and $\hat{\alpha}$ values are repeated below.

First model	Second model	Condition	First model $\hat{\alpha}$	Second model $\hat{\alpha}$	t-statistic	t-test p value	Wilcoxon statistic	Wilcoxon p value
One-stage FDG	Two-stage FDG	E_1 Static	1.95	4.36	-9.5329	2.2×10^{-18}	-17251	5.6×10^{-17}
One-stage FDG	Two-stage FDG	E_1 Dynamic	1.14	2.86	-4.9743	1.3×10^{-6}	-10828	2.1×10^{-7}
One-stage FDG	Two-stage FDG	E_2 No Feedback	1.80	4.16	-0.2676	7.9×10^{-1}	370	7.8×10^{-1}
One-stage FDG	Two-stage FDG	E_2 Self Feedback	2.13	4.48	-0.9389	3.5×10^{-1}	-1270	3.3×10^{-1}
One-stage FDG	Two-stage FDG	E_2 Full Feedback	1.16	2.29	-1.1425	2.5×10^{-1}	-993	4.7×10^{-1}
One-stage FDG	Conformity-2	E_1 Static	1.95	2.81	-15.5364	8.6×10^{-38}	-23271	1.4×10^{-29}
One-stage FDG	Conformity-2	E_1 Dynamic	1.14	1.52	-22.5782	1.1×10^{-60}	-26176	4.2×10^{-36}
One-stage FDG	Conformity-2	E_2 No Feedback	1.80	4.05	-13.5675	4.1×10^{-29}	-13242	1.4×10^{-22}
One-stage FDG	Conformity-2	E_2 Self Feedback	2.13	2.58	-14.2201	9.7×10^{-31}	-12526	4.5×10^{-22}
One-stage FDG	Conformity-2	E_2 Full Feedback	1.16	1.19	-19.5934	4.3×10^{-46}	-14967	5.8×10^{-28}
One-stage FDG	Conformity-4	E_1 Static	1.95	2.88	-15.6584	3.4×10^{-38}	-23255	1.5×10^{-29}
One-stage FDG	Conformity-4	E_1 Dynamic	1.14	1.33	-22.6411	7.0×10^{-61}	-26156	4.8×10^{-36}
One-stage FDG	Conformity-4	E_2 No Feedback	1.80	3.43	-13.6234	2.8×10^{-29}	-13192	1.9×10^{-22}
One-stage FDG	Conformity-4	E_2 Self Feedback	2.13	2.47	-14.7122	3.9×10^{-32}	-12730	9.5×10^{-23}
One-stage FDG	Conformity-4	E_2 Full Feedback	1.16	1.27	-17.2142	1.4×10^{-39}	-14549	1.6×10^{-26}
Two-stage FDG	Conformity-2	E_1 Static	4.36	2.81	-13.7475	7.4×10^{-32}	-21929	1.8×10^{-26}
Two-stage FDG	Conformity-2	E_1 Dynamic	2.86	1.52	-20.7573	5.6×10^{-55}	-25620	1.2×10^{-34}
Two-stage FDG	Conformity-2	E_2 No Feedback	4.16	4.05	-13.0995	9.1×10^{-28}	-13048	5.5×10^{-22}
Two-stage FDG	Conformity-2	E_2 Self Feedback	4.48	2.58	-13.6809	3.3×10^{-29}	-12380	1.3×10^{-21}
Two-stage FDG	Conformity-2	E_2 Full Feedback	2.29	1.19	-18.4389	5.8×10^{-43}	-14821	1.9×10^{-27}
Two-stage FDG	Conformity-4	E_1 Static	4.36	2.88	-13.5041	4.7×10^{-31}	-21619	9.1×10^{-26}
Two-stage FDG	Conformity-4	E_1 Dynamic	2.86	1.33	-20.5675	2.3×10^{-54}	-25558	1.7×10^{-34}
Two-stage FDG	Conformity-4	E_2 No Feedback	4.16	3.43	-13.1662	5.9×10^{-28}	-13068	4.8×10^{-22}
Two-stage FDG	Conformity-4	E_2 Self Feedback	4.48	2.47	-14.0790	2.4×10^{-30}	-12620	2.2×10^{-22}
Two-stage FDG	Conformity-4	E_2 Full Feedback	2.29	1.27	-15.7384	2.1×10^{-35}	-14321	9.7×10^{-26}

Table S16. Model comparisons with subset A data and summary statistic $\widetilde{y}^*_{\text{mean}}$. The best-fitting parameters for these models are given in Table S7, and $\hat{\alpha}$ values are repeated below.

First model	Second model	Condition	First model $\hat{\alpha}$	Second model $\hat{\alpha}$	t-statistic	t-test p value	Wilcoxon statistic	Wilcoxon p value
One-stage FDG	Two-stage FDG	E_1 Static	21.96	35.65	0.6155	5.4×10^{-1}	-15837	1.8×10^{-27}
One-stage FDG	Two-stage FDG	E_1 Dynamic	0.00	23.01	2.4576	1.5×10^{-2}	495	2.4×10^{-1}
One-stage FDG	Two-stage FDG	E_2 No Feedback	5.80	0.23	1.4181	1.6×10^{-1}	78	2.4×10^{-1}
One-stage FDG	Two-stage FDG	E_2 Self Feedback	42.31	31.48	-1.4415	1.5×10^{-1}	291	3.0×10^{-1}
One-stage FDG	Two-stage FDG	E_2 Full Feedback	0.00	0.00	3.3619	9.5×10^{-4}	143	5.4×10^{-2}
One-stage FDG	Conformity-2	E_1 Static	21.96	0.00	-8.3925	4.7×10^{-15}	-6431	2.3×10^{-15}
One-stage FDG	Conformity-2	E_1 Dynamic	0.00	0.00	-8.4342	3.5×10^{-15}	-5952	1.2×10^{-13}
One-stage FDG	Conformity-2	E_2 No Feedback	5.80	0.00	-8.9744	4.4×10^{-16}	-3198	1.7×10^{-14}
One-stage FDG	Conformity-2	E_2 Self Feedback	42.31	0.00	-6.8224	1.5×10^{-10}	-2512	2.1×10^{-8}
One-stage FDG	Conformity-2	E_2 Full Feedback	0.00	0.00	-3.8493	1.7×10^{-4}	-2664	7.6×10^{-7}
One-stage FDG	Conformity-4	E_1 Static	21.96	0.05	-7.9641	7.4×10^{-14}	-6417	2.7×10^{-15}
One-stage FDG	Conformity-4	E_1 Dynamic	0.00	0.01	-8.1820	1.8×10^{-14}	-5876	2.4×10^{-13}
One-stage FDG	Conformity-4	E_2 No Feedback	5.80	0.79	-8.7057	2.3×10^{-15}	-3198	1.7×10^{-14}
One-stage FDG	Conformity-4	E_2 Self Feedback	42.31	0.00	-6.8328	1.4×10^{-10}	-2514	2.1×10^{-8}
One-stage FDG	Conformity-4	E_2 Full Feedback	0.00	0.01	-3.5165	5.6×10^{-4}	-2606	1.3×10^{-6}
Two-stage FDG	Conformity-2	E_1 Static	35.65	0.00	-9.1113	3.9×10^{-17}	-8813	2.7×10^{-7}
Two-stage FDG	Conformity-2	E_1 Dynamic	23.01	0.00	-9.8566	2.2×10^{-19}	-6925	1.4×10^{-17}
Two-stage FDG	Conformity-2	E_2 No Feedback	0.23	0.00	-8.9937	3.9×10^{-16}	-3331	1.4×10^{-14}
Two-stage FDG	Conformity-2	E_2 Self Feedback	31.48	0.00	-6.0350	9.7×10^{-9}	-2175	3.4×10^{-8}
Two-stage FDG	Conformity-2	E_2 Full Feedback	0.00	0.00	-10.0444	4.7×10^{-19}	-4795	2.0×10^{-17}
Two-stage FDG	Conformity-4	E_1 Static	35.65	0.05	-8.8804	1.9×10^{-16}	-8683	4.0×10^{-7}
Two-stage FDG	Conformity-4	E_1 Dynamic	23.01	0.01	-9.7960	3.3×10^{-19}	-6895	2.0×10^{-17}
Two-stage FDG	Conformity-4	E_2 No Feedback	0.23	0.79	-8.7448	1.8×10^{-15}	-3331	1.4×10^{-14}
Two-stage FDG	Conformity-4	E_2 Self Feedback	31.48	0.00	-6.0064	1.1×10^{-8}	-2173	3.5×10^{-8}
Two-stage FDG	Conformity-4	E_2 Full Feedback	0.00	0.01	-9.9788	7.2×10^{-19}	-4795	2.0×10^{-17}

Table S17. Model comparisons with subset A data and summary statistic $\widetilde{y}^*_{\text{median}}$. The best-fitting parameters for these models are given in Table S8, and $\hat{\alpha}$ values are repeated below.

First model	Second model	Condition	First model $\hat{\alpha}$	Second model $\hat{\alpha}$	t-statistic	t-test p value	Wilcoxon statistic	Wilcoxon p value
One-stage FDG	Two-stage FDG	E_1 Static	13.66	27.42	-11.5180	1.4×10^{-24}	-19941	3.7×10^{-22}
One-stage FDG	Two-stage FDG	E_1 Dynamic	10.47	15.99	2.2816	2.3×10^{-2}	3626	8.2×10^{-2}
One-stage FDG	Two-stage FDG	E_2 No Feedback	8.77	16.54	11.1125	4.8×10^{-22}	11790	3.1×10^{-18}
One-stage FDG	Two-stage FDG	E_2 Self Feedback	18.92	41.19	-2.0028	4.7×10^{-2}	-2064	1.1×10^{-1}
One-stage FDG	Two-stage FDG	E_2 Full Feedback	10.68	24.16	-9.2204	9.0×10^{-17}	-10523	1.3×10^{-14}
One-stage FDG	Conformity-2	E_1 Static	13.66	973.43	6.9525	3.6×10^{-11}	13351	9.1×10^{-11}
One-stage FDG	Conformity-2	E_1 Dynamic	10.47	0.00	-5.8567	1.6×10^{-8}	-11416	4.5×10^{-8}
One-stage FDG	Conformity-2	E_2 No Feedback	8.77	2.98	3.7987	2.0×10^{-4}	4914	2.8×10^{-4}
One-stage FDG	Conformity-2	E_2 Self Feedback	18.92	0.94	-1.1147	2.7×10^{-1}	-1340	3.0×10^{-1}
One-stage FDG	Conformity-2	E_2 Full Feedback	10.68	0.01	-6.6025	4.6×10^{-10}	-8057	3.6×10^{-9}
One-stage FDG	Conformity-4	E_1 Static	13.66	773.24	6.2148	2.4×10^{-9}	12051	4.9×10^{-9}
One-stage FDG	Conformity-4	E_1 Dynamic	10.47	0.00	-6.5840	3.0×10^{-10}	-12776	9.2×10^{-10}
One-stage FDG	Conformity-4	E_2 No Feedback	8.77	22.22	3.7851	2.1×10^{-4}	4958	2.5×10^{-4}
One-stage FDG	Conformity-4	E_2 Self Feedback	18.92	0.96	-1.2036	2.3×10^{-1}	-1458	2.6×10^{-1}
One-stage FDG	Conformity-4	E_2 Full Feedback	10.68	0.75	-9.4743	1.8×10^{-17}	-10833	2.1×10^{-15}
Two-stage FDG	Conformity-2	E_1 Static	27.42	973.43	7.2653	5.6×10^{-12}	13961	1.2×10^{-11}
Two-stage FDG	Conformity-2	E_1 Dynamic	15.99	0.00	-6.1480	3.4×10^{-9}	-11990	9.1×10^{-9}
Two-stage FDG	Conformity-2	E_2 No Feedback	16.54	2.98	2.7279	7.0×10^{-3}	3436	1.1×10^{-2}
Two-stage FDG	Conformity-2	E_2 Self Feedback	41.19	0.94	-1.0740	2.8×10^{-1}	-1312	3.1×10^{-1}
Two-stage FDG	Conformity-2	E_2 Full Feedback	24.16	0.01	-6.1728	4.5×10^{-9}	-7607	2.5×10^{-8}
Two-stage FDG	Conformity-4	E_1 Static	27.42	773.24	6.5424	3.8×10^{-10}	12625	8.9×10^{-10}
Two-stage FDG	Conformity-4	E_1 Dynamic	15.99	0.00	-6.8798	5.4×10^{-11}	-13352	1.6×10^{-10}
Two-stage FDG	Conformity-4	E_2 No Feedback	16.54	22.22	2.4129	1.7×10^{-2}	2712	4.5×10^{-2}
Two-stage FDG	Conformity-4	E_2 Self Feedback	41.19	0.96	-1.1626	2.5×10^{-1}	-1422	2.7×10^{-1}
Two-stage FDG	Conformity-4	E_2 Full Feedback	24.16	0.75	-8.9708	4.3×10^{-16}	-10405	2.5×10^{-14}

Table S18. Model comparisons with subset B data and summary statistic $\widetilde{y}^{*}_{\text{sumlogs}}$. The best-fitting parameters for these models are given in Table S9, and $\hat{\alpha}$ values are repeated below.

First model	Second model	Condition	First model $\hat{\alpha}$	Second model $\hat{\alpha}$	t-statistic	t-test p value	Wilcoxon statistic	Wilcoxon p value
One-stage FDG	Two-stage FDG	E_1 Static	1.11	3.21	2.2396	2.8×10^{-2}	1218	1.8×10^{-2}
One-stage FDG	Two-stage FDG	E_1 Dynamic	0.92	2.80	7.3085	3.5×10^{-11}	4590	1.2×10^{-9}
One-stage FDG	Two-stage FDG	E_2 No Feedback	1.75	4.36	8.0525	4.5×10^{-11}	1602	3.7×10^{-9}
One-stage FDG	Two-stage FDG	E_2 Self Feedback	1.30	3.72	-1.7529	8.4×10^{-2}	-524	8.0×10^{-2}
One-stage FDG	Two-stage FDG	E_2 Full Feedback	0.93	2.82	0.2615	7.9×10^{-1}	272	6.4×10^{-1}
One-stage FDG	Conformity-2	E_1 Static	1.11	1.95	-19.4739	3.3×10^{-34}	-4270	9.3×10^{-17}
One-stage FDG	Conformity-2	E_1 Dynamic	0.92	1.57	-22.3621	3.0×10^{-44}	-7120	3.7×10^{-21}
One-stage FDG	Conformity-2	E_2 No Feedback	1.75	2.88	-14.1220	1.4×10^{-20}	-1810	2.7×10^{-11}
One-stage FDG	Conformity-2	E_2 Self Feedback	1.30	1.72	-17.6843	4.1×10^{-26}	-2060	5.7×10^{-12}
One-stage FDG	Conformity-2	E_2 Full Feedback	0.93	1.27	-24.2617	3.2×10^{-43}	-4944	6.2×10^{-18}
One-stage FDG	Conformity-4	E_1 Static	1.11	1.32	-15.5556	2.3×10^{-27}	-4192	3.3×10^{-16}
One-stage FDG	Conformity-4	E_1 Dynamic	0.92	1.68	-22.4767	1.9×10^{-44}	-7112	4.1×10^{-21}
One-stage FDG	Conformity-4	E_2 No Feedback	1.75	2.47	-14.3921	5.8×10^{-21}	-1830	1.6×10^{-11}
One-stage FDG	Conformity-4	E_2 Self Feedback	1.30	1.40	-17.2470	1.5×10^{-25}	-2058	5.9×10^{-12}
One-stage FDG	Conformity-4	E_2 Full Feedback	0.93	1.25	-24.7486	6.0×10^{-44}	-4948	5.9×10^{-18}
Two-stage FDG	Conformity-2	E_1 Static	3.21	1.95	-19.9547	5.4×10^{-35}	-4270	9.3×10^{-17}
Two-stage FDG	Conformity-2	E_1 Dynamic	2.80	1.57	-22.6135	1.0×10^{-44}	-7128	3.4×10^{-21}
Two-stage FDG	Conformity-2	E_2 No Feedback	4.36	2.88	-15.1286	5.7×10^{-22}	-1822	2.0×10^{-11}
Two-stage FDG	Conformity-2	E_2 Self Feedback	3.72	1.72	-16.6604	9.1×10^{-25}	-2054	6.5×10^{-12}
Two-stage FDG	Conformity-2	E_2 Full Feedback	2.82	1.27	-22.6368	1.0×10^{-40}	-4944	6.2×10^{-18}
Two-stage FDG	Conformity-4	E_1 Static	3.21	1.32	-15.8045	7.9×10^{-28}	-4220	2.1×10^{-16}
Two-stage FDG	Conformity-4	E_1 Dynamic	2.80	1.68	-22.6438	9.1×10^{-45}	-7120	3.7×10^{-21}
Two-stage FDG	Conformity-4	E_2 No Feedback	4.36	2.47	-15.4804	1.9×10^{-22}	-1830	1.6×10^{-11}
Two-stage FDG	Conformity-4	E_2 Self Feedback	3.72	1.40	-16.2243	3.5×10^{-24}	-2056	6.2×10^{-12}
Two-stage FDG	Conformity-4	E_2 Full Feedback	2.82	1.25	-22.9309	3.6×10^{-41}	-4946	6.1×10^{-18}

Table S19. Model comparisons with subset B data and summary statistic $\widetilde{y}^{*}_{\mathrm{mean}}$. The best-fitting parameters for these models are given in Table S10, and $\hat{\alpha}$ values are repeated below.

First model	Second model	Condition	First model $\hat{\alpha}$	Second model $\hat{\alpha}$	t-statistic	t-test p value	Wilcoxon statistic	Wilcoxon p value
One-stage FDG	Two-stage FDG	E_1 Static	987.34	996.46	1.9668	5.2×10^{-2}	849	2.2×10^{-2}
One-stage FDG	Two-stage FDG	E_1 Dynamic	0.00	981.43	-1.2510	2.1×10^{-1}	-3204	2.3×10^{-8}
One-stage FDG	Two-stage FDG	E_2 No Feedback	999.93	919.34	2.5913	1.2×10^{-2}	760	1.9×10^{-3}
One-stage FDG	Two-stage FDG	E_2 Self Feedback	996.22	987.81	7.8837	5.7×10^{-11}	1653	5.1×10^{-11}
One-stage FDG	Two-stage FDG	E_2 Full Feedback	0.00	989.11	0.1278	9.0×10^{-1}	-1972	1.4×10^{-6}
One-stage FDG	Conformity-2	E_1 Static	987.34	112.39	-11.3930	3.3×10^{-19}	-3914	3.9×10^{-16}
One-stage FDG	Conformity-2	E_1 Dynamic	0.00	0.00	-7.0352	1.4×10^{-10}	-4835	2.5×10^{-14}
One-stage FDG	Conformity-2	E_2 No Feedback	999.93	251.14	-9.8183	5.1×10^{-14}	-1810	2.7×10^{-11}
One-stage FDG	Conformity-2	E_2 Self Feedback	996.22	0.00	-0.9253	3.6×10^{-1}	-180	5.1×10^{-1}
One-stage FDG	Conformity-2	E_2 Full Feedback	0.00	0.00	-5.5335	2.6×10^{-7}	-3212	4.0×10^{-10}
One-stage FDG	Conformity-4	E_1 Static	987.34	22.06	-11.3763	3.6×10^{-19}	-3914	3.9×10^{-16}
One-stage FDG	Conformity-4	E_1 Dynamic	0.00	19.04	-12.1083	2.0×10^{-22}	-5147	5.0×10^{-16}
One-stage FDG	Conformity-4	E_2 No Feedback	999.93	22.06	-10.4441	4.9×10^{-15}	-1810	2.7×10^{-11}
One-stage FDG	Conformity-4	E_2 Self Feedback	996.22	130.68	-11.5253	3.7×10^{-17}	-1830	1.6×10^{-11}
One-stage FDG	Conformity-4	E_2 Full Feedback	0.00	1.18	-7.7203	1.0×10^{-11}	-3042	3.2×10^{-9}
Two-stage FDG	Conformity-2	E_1 Static	996.46	112.39	-11.2144	7.7×10^{-19}	-3828	5.5×10^{-16}
Two-stage FDG	Conformity-2	E_1 Dynamic	981.43	0.00	-6.6215	1.1×10^{-9}	-3461	7.6×10^{-9}
Two-stage FDG	Conformity-2	E_2 No Feedback	919.34	251.14	-8.9402	1.4×10^{-12}	-1550	2.6×10^{-10}
Two-stage FDG	Conformity-2	E_2 Self Feedback	987.81	0.00	-3.2245	2.0×10^{-3}	-738	6.6×10^{-3}
Two-stage FDG	Conformity-2	E_2 Full Feedback	989.11	0.00	-5.9976	3.4×10^{-8}	-2910	1.5×10^{-8}
Two-stage FDG	Conformity-4	E_1 Static	996.46	22.06	-11.1731	9.3×10^{-19}	-3828	5.5×10^{-16}
Two-stage FDG	Conformity-4	E_1 Dynamic	981.43	19.04	-13.4305	1.6×10^{-25}	-5253	1.8×10^{-18}
Two-stage FDG	Conformity-4	E_2 No Feedback	919.34	22.06	-10.1711	1.4×10^{-14}	-1596	7.5×10^{-11}
Two-stage FDG	Conformity-4	E_2 Self Feedback	987.81	130.68	-11.9864	6.7×10^{-18}	-1830	1.6×10^{-11}
Two-stage FDG	Conformity-4	E_2 Full Feedback	989.11	1.18	-10.4639	1.2×10^{-17}	-3796	1.5×10^{-13}

Table S20. Model comparisons with subset B data and summary statistic $\widetilde{y}^*_{\text{median}}$. The best-fitting parameters for these models are given in Table S11, and $\hat{\alpha}$ values are repeated below.

First model	Second model	Condition	First model $\hat{\alpha}$	Second model $\hat{\alpha}$	t-statistic	t-test p value	Wilcoxon statistic	Wilcoxon p value
One-stage FDG	Two-stage FDG	E_1 Static	10.96	23.01	13.6714	8.8×10^{-24}	4070	2.3×10^{-15}
One-stage FDG	Two-stage FDG	E_1 Dynamic	11.97	28.70	-8.6961	2.4×10^{-14}	-5232	4.0×10^{-12}
One-stage FDG	Two-stage FDG	E_2 No Feedback	17.89	29.15	7.1245	1.7×10^{-9}	1504	3.1×10^{-8}
One-stage FDG	Two-stage FDG	E_2 Self Feedback	12.41	27.21	6.3046	3.2×10^{-8}	1572	1.5×10^{-7}
One-stage FDG	Two-stage FDG	E_2 Full Feedback	0.00	29.54	-1.2609	2.1×10^{-1}	-866	1.3×10^{-1}
One-stage FDG	Conformity-2	E_1 Static	10.96	0.94	-9.6456	1.4×10^{-15}	-3640	1.4×10^{-12}
One-stage FDG	Conformity-2	E_1 Dynamic	11.97	0.79	-12.7024	7.9×10^{-24}	-6570	3.0×10^{-18}
One-stage FDG	Conformity-2	E_2 No Feedback	17.89	1.19	-9.9614	3.0×10^{-14}	-1716	2.7×10^{-10}
One-stage FDG	Conformity-2	E_2 Self Feedback	12.41	0.00	-5.6055	4.9×10^{-7}	-1376	4.2×10^{-6}
One-stage FDG	Conformity-2	E_2 Full Feedback	0.00	1.32	-17.1292	3.1×10^{-31}	-4906	1.1×10^{-17}
One-stage FDG	Conformity-4	E_1 Static	10.96	1.54	-5.7830	1.0×10^{-7}	-2538	7.8×10^{-7}
One-stage FDG	Conformity-4	E_1 Dynamic	11.97	0.86	-10.3855	2.4×10^{-18}	-5956	2.9×10^{-15}
One-stage FDG	Conformity-4	E_2 No Feedback	17.89	0.78	-8.1912	2.6×10^{-11}	-1600	3.9×10^{-9}
One-stage FDG	Conformity-4	E_2 Self Feedback	12.41	1.59	-12.0338	5.6×10^{-18}	-2008	1.9×10^{-11}
One-stage FDG	Conformity-4	E_2 Full Feedback	0.00	1.08	-17.8729	1.3×10^{-32}	-4908	1.1×10^{-17}
Two-stage FDG	Conformity-2	E_1 Static	23.01	0.94	-10.3996	3.7×10^{-17}	-3782	1.8×10^{-13}
Two-stage FDG	Conformity-2	E_1 Dynamic	28.70	0.79	-12.3602	5.1×10^{-23}	-6508	6.2×10^{-18}
Two-stage FDG	Conformity-2	E_2 No Feedback	29.15	1.19	-10.3488	7.0×10^{-15}	-1732	1.8×10^{-10}
Two-stage FDG	Conformity-2	E_2 Self Feedback	27.21	0.00	-5.6913	3.5×10^{-7}	-1394	3.1×10^{-6}
Two-stage FDG	Conformity-2	E_2 Full Feedback	29.54	1.32	-15.3913	6.7×10^{-28}	-4876	1.7×10^{-17}
Two-stage FDG	Conformity-4	E_1 Static	23.01	1.54	-6.8953	6.9×10^{-10}	-2884	2.0×10^{-8}
Two-stage FDG	Conformity-4	E_1 Dynamic	28.70	0.86	-10.0561	1.5×10^{-17}	-5868	7.2×10^{-15}
Two-stage FDG	Conformity-4	E_2 No Feedback	29.15	0.78	-8.5653	6.1×10^{-12}	-1638	1.6×10^{-9}
Two-stage FDG	Conformity-4	E_2 Self Feedback	27.21	1.59	-12.1319	3.9×10^{-18}	-2012	1.7×10^{-11}
Two-stage FDG	Conformity-4	E_2 Full Feedback	29.54	1.08	-15.6642	2.0×10^{-28}	-4880	1.6×10^{-17}

B. Summary and discussion of the above results. In the full (i.e., not subset) data with the summary statistics $\widetilde{y}^*_{\text{sumlogs}}$ and $\widetilde{y}^*_{\text{mean}}$, the conformity model with four free parameters and with two free parameters provided a significantly better fit to the data than either of the two French-DeGroot models for all experimental conditions (Table S12 and Table S13). When the summary statistic was $\widetilde{y}^*_{\text{median}}$ and the full data were analyzed, the results were less straightforward. In some cases, the French-DeGroot models provided a significantly better fit to the data than the conformity models, but in other cases, the opposite was true (Table S14). In all of the former cases, the best-fitting $\hat{\alpha}$ parameters for the conformity and French-DeGroot models were high: greater than 98. As mentioned in Section 1.D of the main text, as α becomes large, the French-DeGroot and conformity models become more similar, making comparisons among them more difficult.

As with the full data, for subset A data with summary statistics $\widetilde{y}^*_{\text{sumlogs}}$ and $\widetilde{y}^*_{\text{mean}}$, both the four- and two-parameter conformity models provided a significantly better fit to the test data than either of the French-DeGroot models in every experimental condition (Table S15 and Table S16). With $\widetilde{y}^*_{\text{median}}$, the conformity models fit the data better than the French-DeGroot models under conditions E_1 Dynamic and E_2 Full Feedback, whereas the opposite was true for conditions E_1 Static and E_2 No Feedback (Table S17). Recall that in both E_1 Dynamic and E_2 Full Feedback, individuals belong to a dynamic network (i.e., they can choose whose answers to view in each round) and receive feedback on the accuracy of their own and others' guesses. Thus, it appears that an individual with the median y^* value tends to conform to the estimates of others when it is possible to choose the latter based on their past estimation accuracy. In E_1 Static, where individuals cannot choose whose answers to view, and E_2 No Feedback, where they do not know whose answers are accurate, an individual with the median y^* value tends to rely more on French-DeGroot averaging. For the condition E_2 Self Feedback, the conformity models provided a slightly better fit to the data than the French-DeGroot models, but the differences were not significant.

As in the full data and subset A data, for subset B data with $\widetilde{y}^*_{\text{sumlogs}}$, the conformity models provided a significantly better fit to the data than either of the French-DeGroot models (Table S18). The same was almost always true when the summary statistic was $\widetilde{y}^*_{\text{mean}}$, but for one condition, shown in Table S19—namely when comparing the one-stage French-DeGroot model to the two-parameter conformity model in the E_2 Self Feedback experiment—the difference was not statistically significant. Finally, when the summary statistic was $\widetilde{y}^*_{\text{median}}$, unlike in the full data and subset A data, the conformity models fit the data better than the French-DeGroot models in all experimental conditions (Table S20).

In summary, we analyzed the full data, subset A data, and subset B data, each with summary statistics $\widetilde{y}^*_{\text{sumlogs}}$, $\widetilde{y}^*_{\text{mean}}$, and $\widetilde{y}^*_{\text{median}}$. For each case, we performed 20 statistical analyses comparing the French-DeGroot and conformity models' fit with the test data (2 versions of the French-DeGroot model $\times$ 2 versions of the conformity model $\times$ 5 experimental conditions). In the full and subset A data with $\widetilde{y}^*_{\text{sumlogs}}$ and $\widetilde{y}^*_{\text{mean}}$, the conformity models outperformed the French-DeGroot models in all 20 of these cases. The results were more mixed with $\widetilde{y}^*_{\text{median}}$. In subset B data with $\widetilde{y}^*_{\text{sumlogs}}$, $\widetilde{y}^*_{\text{mean}}$, and $\widetilde{y}^*_{\text{median}}$, the conformity models outperformed the French-DeGroot models in 20, 19, and 20 of the 20 conditions, respectively.

4. Evolutionary simulations

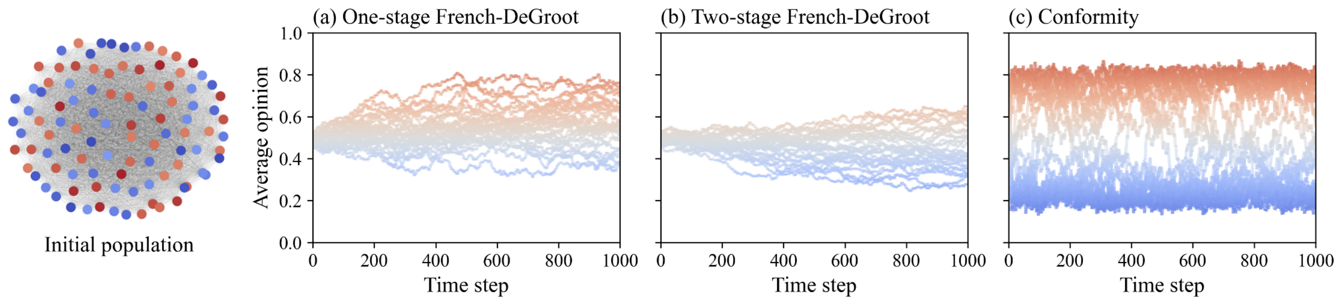

Fig. S1. Similar to Figure 2 in the main text, but with a different distribution of initial opinions in the population. The initial opinions of $N = 50$ members of the population are drawn from a uniform distribution on $[0, 0.2]$ and the initial opinions of the other $N = 50$ members of the population are drawn from a uniform distribution on $[0.8, 1]$.

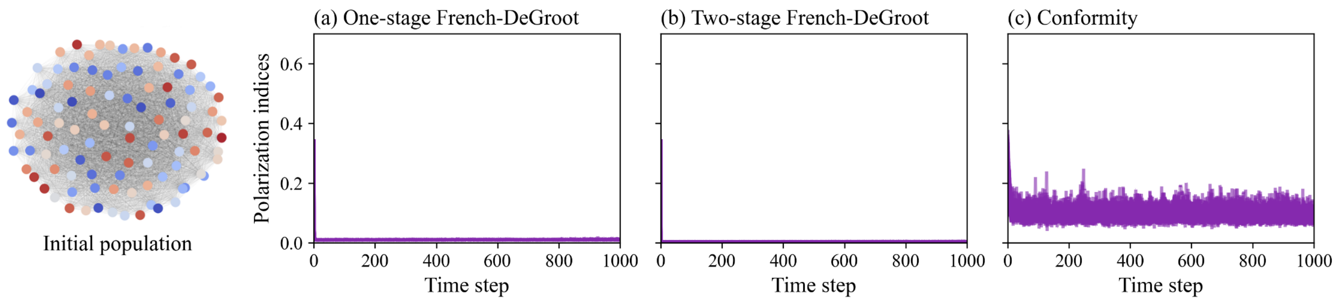

Fig. S2. These simulations correspond to Figure 2 in the main text, but here, rather than the average opinions in the population over time (as in Figure 2), the polarization indices F are plotted over time. F is defined in (4) and in the main text.

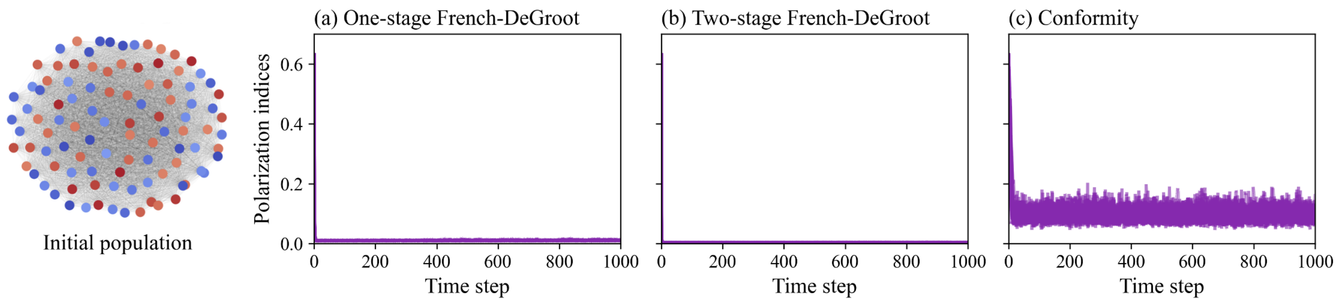

Fig. S3. These simulations correspond to Figure S1, but rather than the average opinions in the population over time, the polarization indices F are plotted over time.

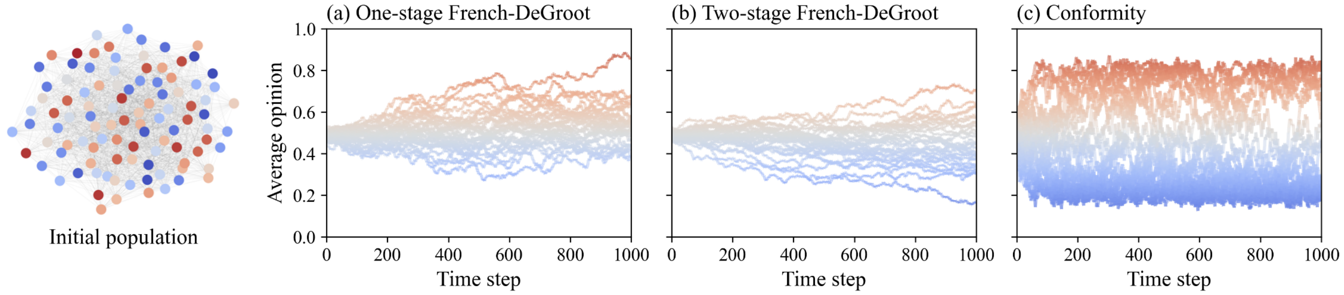

Fig. S4. Evolutionary simulation results for $N = 100$ individuals in a static network. Each individual's initial opinion is drawn from a uniform distribution on $[0, 1]$. More pronounced blue colors correspond to opinions closer to 0 and more pronounced red colors correspond to opinions closer to 1. Panels (a-c) show the dynamics under the one-stage French-DeGroot model, two-stage French-DeGroot model, and conformity model, respectively. Each plot includes 30 replicates of the model, and shows the average opinion in the population for each of these replicates for 1000 time steps. The average opinion is colored more blue as it approaches 0 and more red as it approaches 1. The parameters of the model are $n = 3$, $\sigma = 0.08$, $\alpha = 2$, $d = 0.8$, and $k = 2$, as in Figure 1.

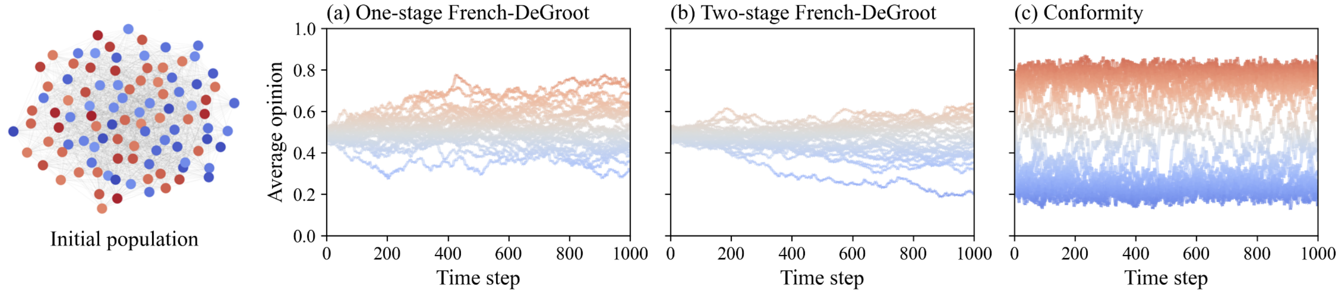

Fig. S5. Similar to Figure S4, but with a different distribution of initial opinions in the population. The initial opinions of $N = 50$ members of the population are drawn from a uniform distribution on $[0, 0.2]$ and the initial opinions of the other $N = 50$ members of the population are drawn from a uniform distribution on $[0.8, 1]$.

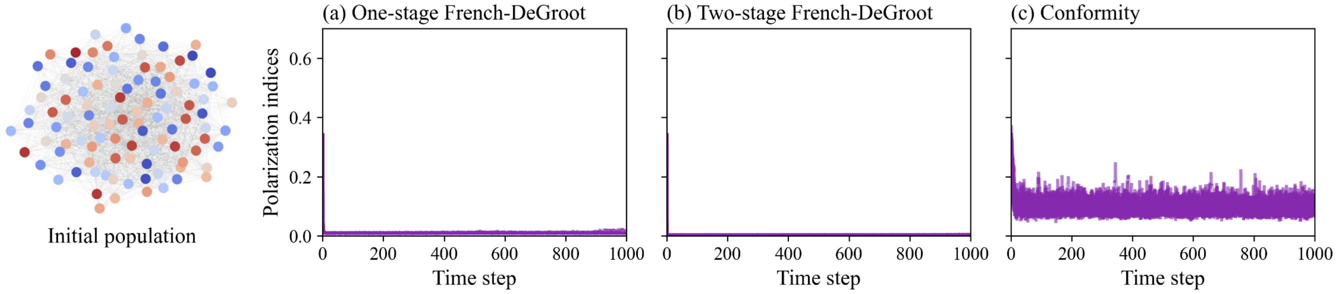

Fig. S6. These simulations correspond to Figure S4, but rather than the average opinions in the population over time, the polarization indices F are plotted over time.

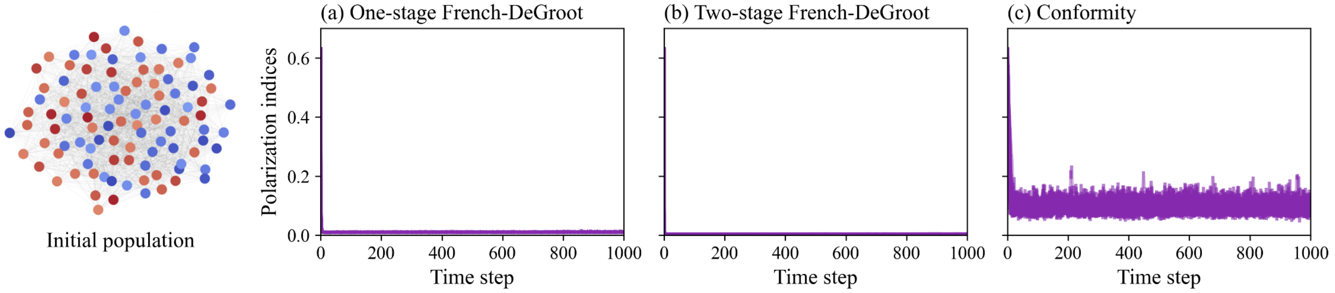

Fig. S7. These simulations correspond to Figure S5, but rather than the average opinions in the population over time, the polarization indices F are plotted over time.

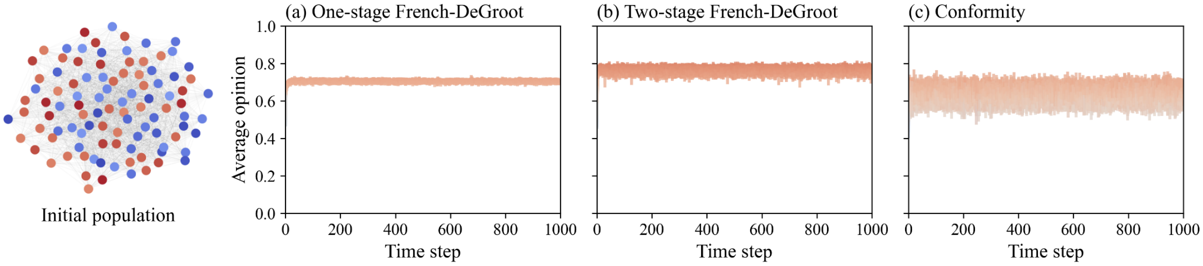

Fig. S8. Similar to Figure 3 in the main text, but with a different distribution of initial opinions in the population. The initial opinions of $N = 50$ members of the population are drawn from a uniform distribution on $[0, 0.2]$ and the initial opinions of the other $N = 50$ members of the population are drawn from a uniform distribution on $[0.8, 1]$.

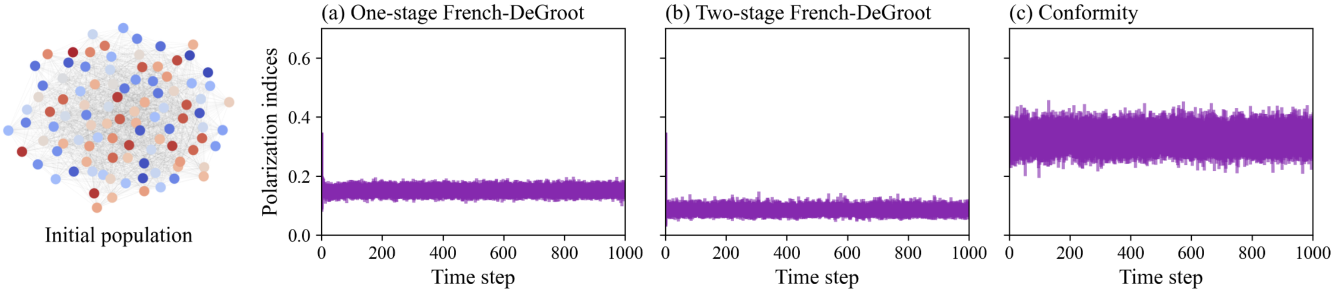

Fig. S9. These simulations correspond to Figure 3 in the main text, but rather than the average opinions in the population over time, the polarization indices F are plotted over time.

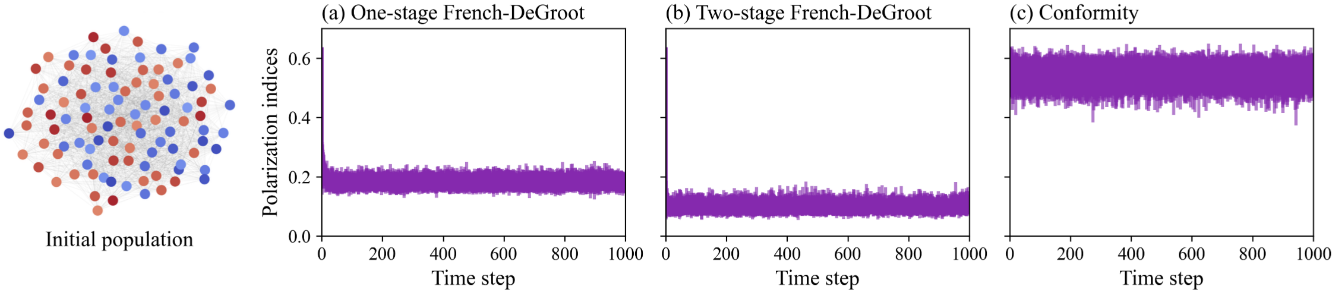

Fig. S10. These simulations correspond to Figure S8, but rather than the average opinions in the population over time, the polarization indices F are plotted over time.

References

1. A Almaatouq, et al., Adaptive social networks promote the wisdom of crowds. *Proc. Natl. Acad. Sci. USA* **117**, 11379–11386 (2020).
2. J Bergstra, D Yamins, D Cox, Making a science of model search: Hyperparameter optimization in hundreds of dimensions for vision architectures in *International conference on machine learning.* (PMLR), pp. 115–123 (2013).
3. J Bergstra, R Bardenet, Y Bengio, B Kégl, Algorithms for hyper-parameter optimization. *Adv. neural information processing systems* **24** (2011).
4. O Leimar, SRX Dall, AI Houston, JM McNamara, Behavioural specialization and learning in social networks. *Proc. Royal Soc. B* **289**, 20220954 (2022).
